# ZCCHC3 is a co-sensor of cGAS for dsDNA recognition in innate immune response

**DOI:** 10.1038/s41467-018-05559-w

**Published:** 2018-08-22

**Authors:** Huan Lian, Jin Wei, Ru Zang, Wen Ye, Qing Yang, Xia-Nan Zhang, Yun-Da Chen, Yu-Zhi Fu, Ming-Ming Hu, Cao-Qi Lei, Wei-Wei Luo, Shu Li, Hong-Bing Shu

**Affiliations:** 1 Medical Research Institute, School of Medicine, Wuhan University, 430071 Wuhan, China; 2 College of Life Sciences Wuhan University, 430072 Wuhan, China; 3 Wuhan Institute of Virology, Chinese Academy of Sciences, 430071 Wuhan, China

**Keywords:** Antimicrobial responses, Innate immunity, Viral host response, Infection

## Abstract

Cyclic GMP-AMP synthase (cGAS) senses double-strand (ds) DNA in the cytosol and then catalyzes synthesis of the second messenger cGAMP, which activates the adaptor MITA/STING to initiate innate antiviral response. How cGAS activity is regulated remains enigmatic. Here, we identify ZCCHC3, a CCHC-type zinc-finger protein, as a positive regulator of cytosolic dsDNA- and DNA virus-triggered signaling. We show that ZCCHC3-deficiency inhibits dsDNA- and DNA virus-triggered induction of downstream effector genes, and that ZCCHC3-deficient mice are more susceptible to lethal herpes simplex virus type 1 or vaccinia virus infection. ZCCHC3 directly binds to dsDNA, enhances the binding of cGAS to dsDNA, and is important for cGAS activation following viral infection. Our results suggest that ZCCHC3 is a co-sensor for recognition of dsDNA by cGAS, which is important for efficient innate immune response to cytosolic dsDNA and DNA virus.

## Abstract (web summary)

cGAS is an important mediator of antiviral immune responses, but the regulation of its activity is unknown. Here, the authors identify a zinc finger protein, ZCCHC3, that enhances the binding of cGAS to dsDNA and is important for its activation following viral infection.

## Introduction

The innate immune system is the first line of host defense against microbial infection. Upon microbial infection, cellular pattern recognition receptors (PRRs) recognize structurally conserved microbial components called pathogen-associated molecular patterns (PAMPs), which triggers a series of signaling events that lead to the induction of type I interferons (IFNs), pro-inflammatory cytokines and other downstream effectors. These downstream effectors mediate the inhibition of microbial replication, clearance of infected cells and facilitation of adaptive immune response to eliminate infected pathogens^1–5^.

Microbial nucleic acids are major PAMPs that are sensed by cellular PRRs after microbial infection. Among identified PRRs, endosomal Toll-like receptor 3 (TLR3) and retinoic acid-inducible gene-I (RIG-I)-like receptors (RLRs), including RIG-I and MDA5, play important roles in recognition of viral RNA^1^. Previously, it has been shown that several DNA sensors including Sox2^6^, TLR9^7^, AIM2^8^, DAI^9^, RNA polymerase III^10,11^, IFI16^12^, DDX41^13^, and LSm14A^14^ can detect cytosolic or microbial DNA in distinct cells or mouse models. However, these proteins are not universally required for cytosolic DNA sensing in distinct cell types or in vivo^5^. A nucleotidyltransferase family member, called cyclic GMP-AMP (cGAMP) synthase (cGAS), has been defined as a key DNA sensor in various cell types and mice^15^. It has been demonstrated that cGAS recognizes cytosolic DNAs derived from various types of virus, including DNA viruses (such as herpes virus, adenovirus, and hepatitis B virus) and retroviruses (such as HIV-1)^16–19^. cGAS also detects other pathogenic DNA and mitochondrial DNA in the cytosol^20–22^. Genetic studies have demonstrated that cGAS plays crucial roles in innate immune responses to cytosolic DNA and various DNA viruses^23^.

After recognition of dis-located cellular or microbial DNA in the cytosol, cGAS undergoes oligomerization and catalyzes the synthesis of the second messenger molecule cGAMP from ATP and GTP^24,25^, which in turn binds to and activates the adaptor MITA (also known as STING, MPYS, and ERIS) located in the endoplasmic reticulum (ER)^26–30^. MITA then translocates from the ER via ER-Golgi intermediate compartments and Golgi apparatus to perinuclear punctuate structures. During the trafficking processes, MITA recruits the kinaseTBK1 and the transcription factor IRF3, leading to their phosphorylation and activation as well as induction of downstream effector genes^31,32^.

Structural studies have revealed certain modes on how cGAS recognizes DNA. cGAS contains an N-terminal domain (aa1-160) with an unknown function and a C-terminal Mab21 domain (aa161-522) that belongs to the nucleotidyltransferase (NTase) superfamily. The N-terminal part of the Mab21 domain is a NTase fold (aa148-370) that is important for sensing of DNA and synthesis of cGAMP^15,33^. Upon DNA binding, cGAS forms a 2:2 dimer (composed of two cGAS and two DNA molecules) or higher-order complexes, leading to the activation of cGAS^34,35^. Recent studies have also demonstrated that longer DNA ligands activate cGAS at a higher rate than shorter DNA, and the formation of stable cGAS_2*n*_-DNA_2_ ladders (*n* ≧ 2) is important for activation of cGAS^36^. However, it has been shown that the affinity of cGAS for DNA is low and cGAS can recognize DNA with distinct properties^33,35,37^, which are in contrast with its ability to specifically respond to small quantities of microbial and self DNA in the cytosol. Recently, it has been shown that the cellular protein PQBP1 serves as a co-sensor by directly binding to single-strand (ss) reverse-transcribed HIV-1 DNA and interacting with cGAS to initiate innate immune response. However, PQBP1 is not required for cGAS-dependent responses to all cytosolic double-strand (ds) DNA but only the ssDNA derived from retroviral infection^38^. Whether cGAS requires co-sensors analogous to PQBP1 to detect non-retroviral cytoplasmic dsDNA is still an unanswered question in the field. In this study, we identified the CCHC-type zinc-finger (ZF) protein ZCCHC3 as a co-sensor of cGAS in innate immune response to cytosolic dsDNA and DNA virus. Deficiency of ZCCHC3 impaired cytosolic dsDNA- or DNA virus-triggered induction of type I IFNs, pro-inflammatory cytokines and other effectors in cells and mice. *Zcchc3*^−*/*−^ mice were more susceptible to lethal infection by DNA viruses. Mechanistically, ZCCHC3 bound to dsDNA, promoted the binding of cGAS to dsDNA, and was important for the enzymatic activity of cGAS following dsDNA stimulation or DNA virus infection. Our findings suggest that ZCCHC3 acts as a co-sensor of cGAS for recognition of dsDNA and plays an essential role in innate immune response to DNA virus.

## Results

### ZCCHC3 positively regulates dsDNA-triggered signaling

**Fig. 1:**
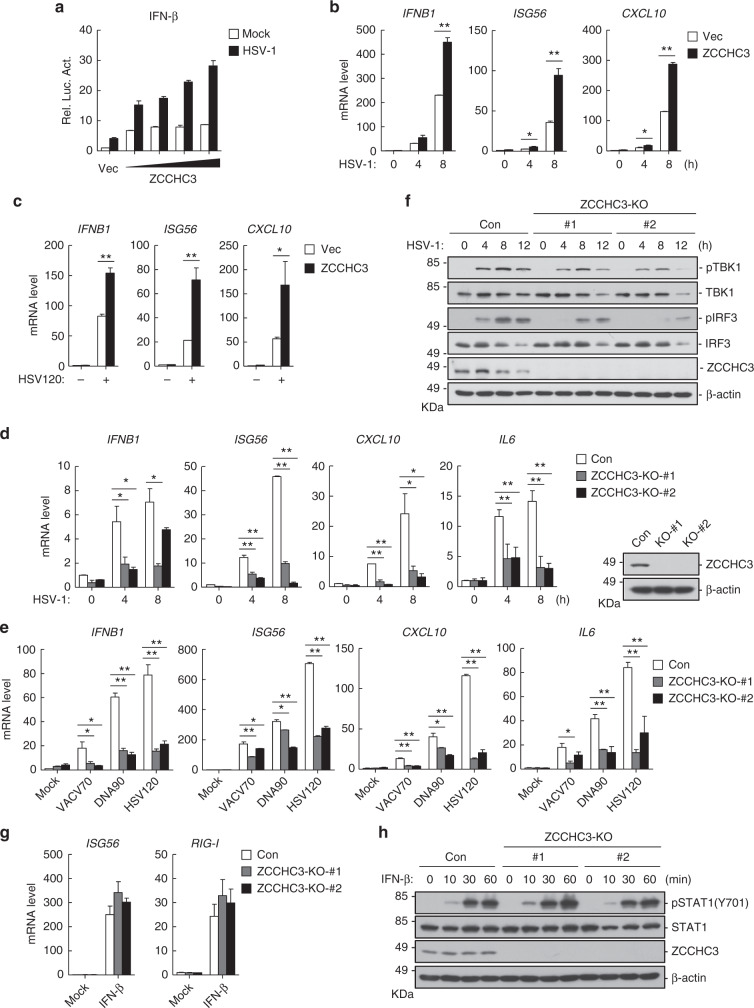
ZCCHC3 positively regulates dsDNA-triggered signaling. **a** ZCCHC3 activates the IFN-β promoter in a dose-dependent manner. HEK293 cells were transfected with the IFN-β reporter and increased amounts of ZCCHC3 plasmid for 18 h, and then left un-infected or infected with HSV-1 for 12 h before luciferase assays. **b** Effects of ZCCHC3 on transcription of downstream genes induced by HSV-1. Control HFF cells and HFFs stably expressing ZCCHC3 were infected with HSV-1 for the indicated times before qPCR analysis. **c** Effects of ZCCHC3 on transcription of downstream genes induced by cytosolic dsDNA. Control and HFFs stably expressing ZCCHC3 were transfected with HSV120 (3 μg/ml) for 4 h before qPCR analysis. **d** Effects of ZCCHC3-deficiency on transcription of downstream genes induced by HSV-1. ZCCHC3- KO HFFs were generated by the CRISPR-Cas9 method. ZCCHC3-KO and control HFFs were left un-infected or infected with HSV-1 for the indicated times before qPCR analysis. ZCCHC3-deficiency in the KO cells was confirmed by immunoblotting analysis (right blots). **e** Effects of ZCCHC3 on transcription of downstream genes induced by cytosolic dsDNA. ZCCHC3-KO and control HFFs were transfected with the indicated nucleic acids (3 μg/ml) for 4 h before qPCR analysis. **f** Effects of ZCCHC3-deficiency on HSV-1-induced phosphorylation of TBK1 and IRF3. ZCCHC3-KO and control HFFs were left un-infected or infected with HSV-1 for the indicated times before immunoblotting analysis. **g** Effects of ZCCHC3-deficiency on IFN-β-induced transcription of downstream genes. Control and ZCCHC3- KO THP1 cells were generated by the CRISPR-Cas9 method. The cells were left un-treated or treated with IFN-β (100 ng/ml) for 6 h before qPCR analysis. **h** Effects of ZCCHC3-deficiency on IFN-β-induced phosphorylation of STAT1. ZCCHC3-KO and control THP1 cells were left un-treated or treated with IFN-β (100 ng/ml) for the indicated times before immunoblotting analysis. * P < 0.05, ** P < 0.01 (unpaired *t* test). Data are representative of at least two experiments with similar results (mean ± SD, *n* = 3 independent samples in **a**–**e**, **g**)

To identify candidate molecules involved in innate immune response, we screened ~10,000 independent human cDNA clones for their abilities to regulate IFN-stimulated response element (ISRE) activation by reporter assays and identified ZCCHC3 as a candidate protein. Reporter assays indicated that overexpression of ZCCHC3 activated the IFN-β promoter (which is driven by ISRE and κB enhancers) by itself and potentiated herpes simplex virus 1 (HSV-1)-induced activation of the IFN-β promoter in a dose-dependent manner in HEK293 cells (Fig. 1a). qPCR experiments indicated that overexpression of ZCCHC3 potentiated HSV-1-induced transcription of *IFNB1*, *ISG56* and *CXCL10* genes in primary human foreskin fibroblasts (HFFs) (Fig. 1b). Previously, it has been shown that transfected dsDNAs, such as the 120-mer dsDNA representing the genome of HSV-1 (HSV120), dsDNA of ~90 bp (dsDNA90), 70-mer dsDNA representing vaccinia virus (VACV) genome (VACV70), and 45-mer IFN stimulatory DNA (ISD45) can efficiently induce transcription of downstream effector genes^28,39^. As shown in Fig. 1c, overexpression of ZCCHC3 potentiated transcription of *IFNB1*, *ISG56* and *CXCL10* genes induced by transfected HSV120 in HFFs. These results suggest that ZCCHC3 is involved in cytosolic dsDNA- and DNA virus-triggered induction of downstream antiviral genes.

To determine whether endogenous ZCCHC3 is important for dsDNA-triggered signaling, we generated ZCCHC3-deficient HFFs and THP1 cells by the CRISPR-Cas9 method. qPCR analysis indicated that knockout of ZCCHC3 dramatically inhibited HSV-1- and transfected dsDNA-induced transcription of downstream genes such as *IFNB1*, *ISG56*, *CXCL10*, and *IL6* in HFFs (Fig. 1d, e). In addition, the phosphorylation of TBK1 and IRF3 induced by HSV-1 in ZCCHC3-deficient HFFs was markedly inhibited compared with control cells (Fig. 1f). Similarly, the transcription of downstream genes and phosphorylation of TBK1and IRF3 induced by HSV-1 in ZCCHC3-deficient THP1 cells was markedly inhibited compared with control cells (Supplementary Fig. 1). In contrast, IFN-β-induced transcription of *ISG56* and *RIG-I* and the phosphorylation of STAT1(Y701) was comparable between ZCCHC3-deficient and control THP1 cells (Fig. 1g, h). These results suggest that ZCCHC3 is an important component in cytosolic dsDNA- and DNA virus-triggered induction of downstream effector genes.

### ZCCHC3 is essential for innate immune response to DNA virus in mice

**Fig. 2:**
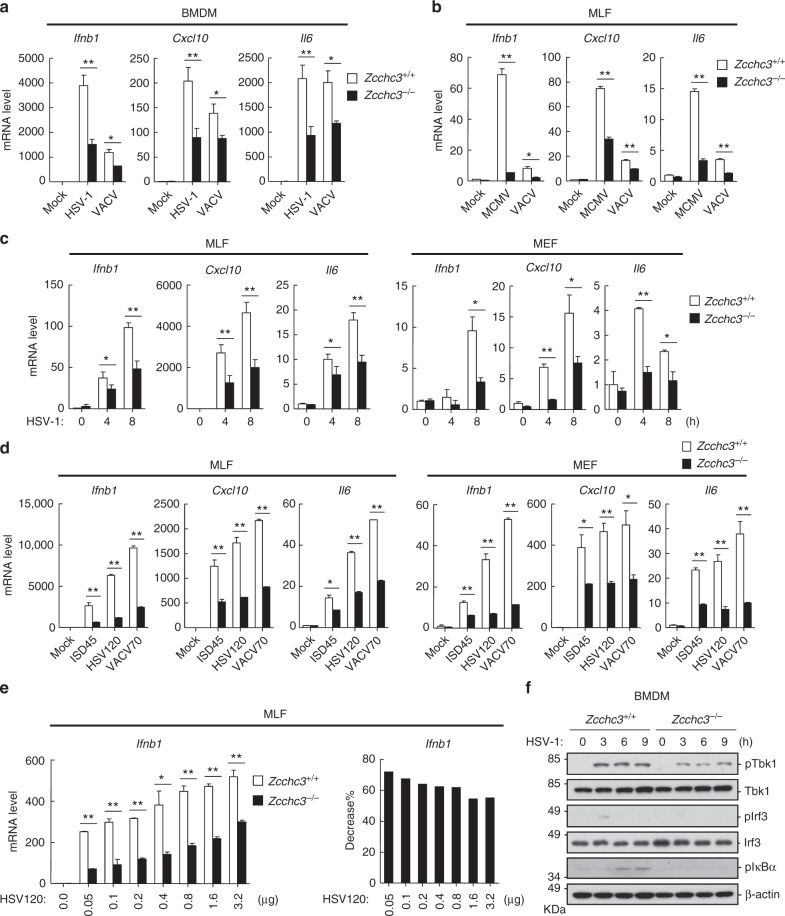
ZCCHC3 is required for DNA virus- or dsDNA-triggered induction of downstream genes. **a** Effects of ZCCHC3-deficiency on HSV-1- or VACV-induced transcription of downstream genes in BMDMs. *Zcchc3*^*+/+*^ and *Zcchc3*^−*/*−^ BMDMs were left un-infected or infected with HSV-1 or VACV for 6 h before qPCR analysis. **b** Effects of ZCCHC3-deficiency on MCMV- or VACV-induced transcription of downstream genes in MLFs. *Zcchc3*^*+/+*^ and *Zcchc3*^−*/*−^ MLFs were left un-infected or infected with MCMV or VACV for 6 h before qPCR analysis. **c** Effects of ZCCHC3-deficiency on HSV-1-induced transcription of downstream genes in MLFs and MEFs. *Zcchc3*^*+/+*^ and *Zcchc3*^−*/*−^ MLFs or MEFs were left un-infected or infected with HSV-1 for the indicated times before qPCR analysis. **d** Effects of ZCCHC3-deficiency on transcription of downstream genes induced by transfected dsDNAs. *Zcchc3*^*+/+*^ and *Zcchc3*^−*/*−^ MLFs or MEFs were transfected with the indicated dsDNAs for 4 h before qPCR analysis. **e** Effects of ZCCHC3-deficiency on transcription of *Ifnb1*gene induced by different doses of HSV120. *Zcchc3*^*+/+*^ and *Zcchc3*^−*/*−^ MLFs were transfected with increased amounts of HSV120 for 4 h before qPCR analysis. The percentage reduction transfection of *Ifnb1* in *Zcchc3*^−*/*−^ compared with *Zcchc3*^*+/+*^ MLFs was shown in the right panels. **f** Effects of ZCCHC3-deficiency on HSV-1-induced phosphorylation of TBK1, IRF3, and ΙκΒα. *Zcchc3*^*+/+*^ and *Zcchc3*^−*/*−^ BMDMs were left un-infected or infected with HSV-1 for the indicated times before immunoblotting analysis. * P < 0.05, ** P < 0.01 (unpaired *t* test). Data are representative of at least two experiments with similar results (mean ± SD, *n* = 3 independent samples in **a**–**e**)

To further investigate the roles of ZCCHC3 in vivo, we generated *Zcchc3* knockout mice. qPCR analysis indicated that transcription of downstream genes including *Ifnb1*, *Cxcl10*, and *Il6* following infection with HSV-1 and VACV was markedly inhibited in *Zcchc*3^−/−^ mouse bone marrow-derived macrophages (BMDMs) (Fig. 2a) and dendrite cells (BMDCs) (Supplementary Fig. 2A). Consistently, ZCCHC3-deficiency dramatically inhibited murine cytomegalovirus (MCMV)-, VACV-, or HSV-1-induced transcription of downstream genes in mouse lung fibroblasts (MLFs) and embryonic fibroblasts (MEFs) (Fig. 2b, c). In addition, transcription of downstream genes such as *Ifnb1*, *Cxcl10*, and *Il6* following transfection of the synthetic dsDNA, ISD45, HSV120, and VACV70, was inhibited in BMDCs (Supplementary Fig. 2B), MLFs and MEFs (Fig. 2d), suggesting that ZCCHC3 has a general role in dsDNA-triggered signaling in distinct cell types. We further investigated the transcription of *Ifnb1* gene induced by transfection of increased amounts of HSV120 in *Zcchc3*^−*/*−^ and *Zcchc3*^*+/+*^ MLFs. The results indicated that HSV120-induced transcription of *Ifnb1* gene was inhibited better in *Zcchc3*^−*/*−^ compared with *Zcchc3*^*+/+*^ MLFs when lower doses of DNA were transfected (Fig. 2e), suggesting that ZCCHC3 plays a more important modulatory role on cGAS activation when cytosolic dsDNA concentration is low.

Further experiments indicated that the secretion of IFN-β and IL-6 were markedly decreased in *Zcchc3*^−*/*−^ BMDCs following HSV-1 infection (Supplementary Fig. 2C) or transfection of ISD45 and HSV120 (Supplementary Fig. 2D). Consistently, phosphorylation of TBK1, IRF3, and IκBα induced by HSV-1 was inhibited in *Zcchc3*^−*/*−^ in comparison to wild-type BMDMs (Fig. 2f) and BMDCs (Supplementary Fig. 2E). In contrast, transcription of *Isg56* and *Rig-I* genes triggered by IFN-β was comparable between *Zcchc3*^−*/*−^ and *Zcchc3*^*+/+*^ MLFs (Supplementary Fig. 2F). Collectively, these data suggest that ZCCHC3 modulates cytosolic dsDNA- and DNA virus-triggered induction of downstream antiviral genes in primary mouse immune cells and fibroblasts.

**Fig. 3:**
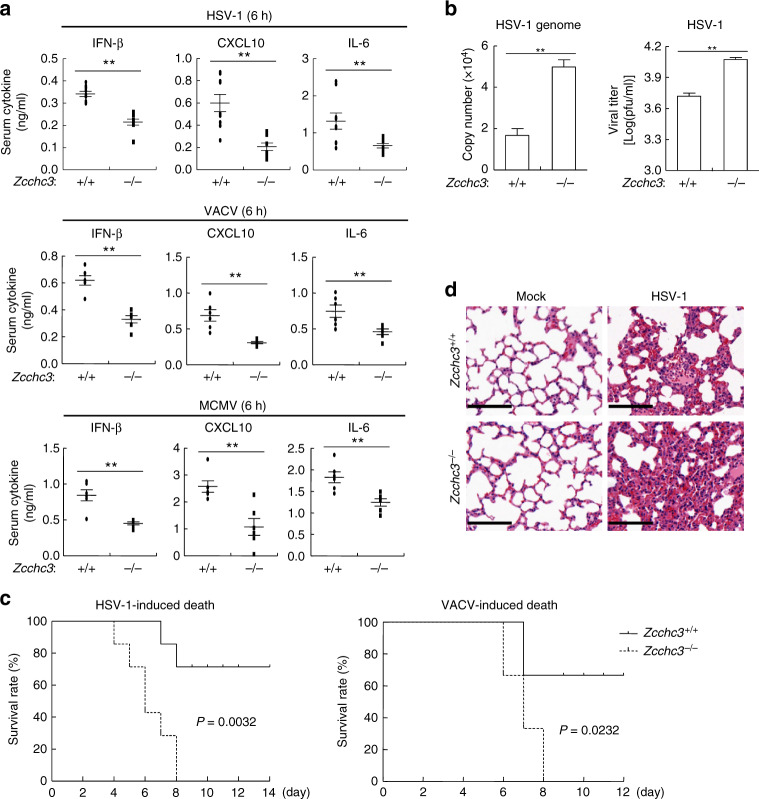
ZCCHC3 is essential for host defense against DNA virus in mice. **a** Effects of ZCCHC3-deficiency on serum levels of IFN-β, CXCL10, and IL-6 induced by DNA viruses. *Zcchc3*^*+/+*^ and *Zcchc3*^−*/*−^ mice (*n* *=* 6–9 per strain, 8 weeks old) were infected with HSV-1, VACV or MCMV (3 × 10^7^, 5 × 10^6^, and 1 × 10^4^ PFU per mouse respectively) for 6 h before measurement of the indicated serum cytokines by ELISA. Each symbol represents an individual mouse. **b** Measurement of viral genomic copy numbers and viral titers. *Zcchc3*^*+/+*^ and *Zcchc3*^−*/*−^ mice (*n* *=* *3* per strain, 8 weeks old) were infected with HSV-1 at 3 × 10^7^ PFU per mouse for 6–7 days. HSV-1 genomic copy numbers and viral titers in the brains of infected mice were quantified by qPCR and plaque assays respectively. **c** Effects of ZCCHC3-deficiency on HSV-1-or VACV-induced death of mice. *Zcchc3*^*+/+*^ and *Zcchc3*^−*/*−^ mice (*n* *=* *7* per strain for HSV-1, *n* *=* *6* per strain for VACV, 8 weeks old) were intra-nasally infected with HSV-1 at 3 × 10^7^ or VACV at 5 × 10^6^ PFU per mouse, and the survival rates of mice were monitored daily. **d** Effects of ZCCHC3-deficiency on HSV-1-induced inflammation in the lungs of mice. Sex and age-matched *Zcchc3*^*+/+*^ and *Zcchc3*^−*/*−^ mice (*n* *=* *3* per strain, 8 weeks old) were intra-nasally infected with HSV-1 at 3 × 10^7^ PFU per mouse for 6–7 days and lung sections were analyzed by H&E staining. Scale bars, 100 μm. *P < 0.05, **P < 0.01 (unpaired *t* test (**a**, **b**) or log-rank test (**c**). Data are representative of at least two experiments with similar results

We next determined the importance of ZCCHC3 in host defense against viral infection in vivo. We found that induction of serum cytokines including IFN-β, CXCL10, and IL-6 following infection of the DNA viruses HSV-1, VACV, and MCMV was severely impaired in *Zcchc3*^−*/*−^ in comparison to *Zcchc3*^*+/+*^ mice (Fig. 3a). Given that HSV-1 is a neurotropic virus and the leading cause of sporadic viral encephalitis, we intra-nasally infected *Zcchc3*^*+/+*^ and *Zcchc3*^−*/*−^ mice with HSV-1 and examined HSV-1 genomic copy numbers and viral titers in the brains. The results indicated that the amounts of HSV-1 genomic copy numbers and viral titers in the brains were much higher in *Zcchc3*^−*/*−^ than *Zcchc3*^*+/+*^ mice at 6 days after viral infection (Fig. 3b). In addition, we found that *Zcchc3*^−*/*−^ mice were more susceptible to HSV-1-or VACV- induced death (Fig. 3c). Consistently, the lungs of *Zcchc3*^−*/*−^ mice showed greater infiltration of immune cells and tissue damage compared to that of *Zcchc3*^*+/+*^mice after HSV-1 infection (Fig. 3d). These results indicated that ZCCHC3 is essential for host defense against DNA virus in vivo.

### ZCCHC3 interacts with cGAS

**Fig. 4:**
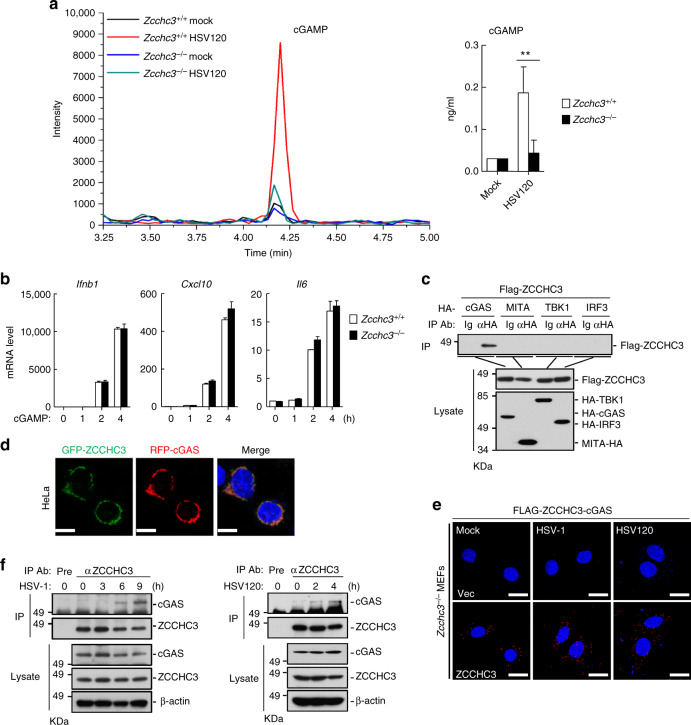
ZCCHC3 acts at the level of cGAS. **a** Effects of ZCCHC3-deficiency on cGAMP synthesis induced by transfected HSV120. *Zcchc3*^*+/+*^ and *Zcchc3*^−*/*−^ MLFs were left untreated or treated with HSV120 (3 μg/ml) for 4 h, and then cell extracts containing cGAMP were separated by chromatography using a C18 column, the abundance of cGAMP was quantitated by mass spectrometry using SRM. The concentration of cGAMP (ng/ml) in *Zcchc3*^*+/+*^ and *Zcchc3*^−*/*−^ MLFs was shown in the right histograph (mean ± SD, *n* = 3 technical repeats, **P < 0.01. **b** Effects of ZCCHC3-deficiency on transcription of downstream genes induced by cGAMP. *Zcchc3*^*+/+*^ and *Zcchc3*^−*/*−^ MLFs were left un-treated or treated with 2′3′-cGAMP (0.2 mg/ml) for 4 h before qPCR analysis. **c** ZCCHC3 is associated with cGAS. HEK293 cells were transfected with the indicated plasmids before co-immunoprecipitation and immunoblotting analysis. **d** Colocalization of ZCCHC3 with cGAS. HeLa cells were transfected with GFP-ZCCHC3 and RFP-cGAS for 20 h before confocal microscopy. Scale bars, 10 μm. **e** PLA analysis of ZCCHC3 and cGAS interaction. *Zcchc3*^−*/*−^ MEFs reconstituted with an empty vector or Flag-ZCCHC3 were infected with HSV-1 or transfected with HSV120 (3 μg/ml) for 4 h before PLA analysis. Scale bars, 20 μm. **f** Endogenous ZCCHC3 is associated with cGAS in THP1 cells. THP1 cells were left un-infected or infected with HSV-1or transfected with HSV120 (3 μg/ml) for the indicated times before co-immunoprecipitation and immunoblotting analysis. Data are representative of at least two experiments with similar results

We next investigated the mechanisms on how ZCCHC3 is involved in cytosolic dsDNA-triggered signaling. Quantitative mass spectrometry showed that the levels of cGAMP induced by transfection of HSV120 was markedly reduced in *Zcchc3*^−*/*−^ in comparison to *Zcchc3*^*+/+*^ MLFs (Fig. 4a), suggesting that ZCCHC3 is essential for dsDNA-induced catalytic activity of cGAS. In contrast, ZCCHC3-deficiency had no effects on cGAMP-induced transcription of downstream genes such as *Ifnb1*, *Cxcl10*, and *Il6* (Fig. 4b). These results suggest that ZCCHC3 acts upstream of cGAMP.

We next determined whether ZCCHC3 is associated with signaling components in dsDNA-triggered signaling pathways. Co-immunoprecipitation experiments indicated that ZCCHC3 was associated with cGAS but not MITA, TBK1, or IRF3 in mammalian overexpression system (Fig. 4c). Confocal microscopy indicated that ZCCHC3 was co-localized with cGAS in the cytoplasm (Fig. 4d). Furthermore, proximity ligation assay (PLA) showed that the co-localization of ZCCHC3 and cGAS in the cytoplasm was increased after HSV-1 infection or HSV120 transfection in MEFs (Fig. 4e). Consistently, endogenous co-immunoprecipitation experiments indicated that ZCCHC3 was barely associated with cGAS in un-infected cells and their association was increased after HSV-1 infection or HSV120 transfection (Fig. 4f).

To further study which domains of ZCCHC3 and cGAS are involved in their interaction, we constructed a series of truncation mutants of ZCCHC3 and cGAS. Domain mapping experiments indicated that the NTase fold (aa213-382) and the C-terminal fragment (aa383-522) of cGAS could independently interact with ZCCHC3 (Supplementary Fig. 3A). On the other hand, the C-terminal ZF domains (aa300-404) of ZCCHC3 interacted very weakly while its N-terminus failed to interact with cGAS, suggesting that the full-length protein of ZCCHC3 is important for its efficient interaction with cGAS (Supplementary Fig. 3B). Taken together, these results suggest that ZCCHC3 targets cGAS after viral infection.

### ZCCHC3 acts as a co-sensor of cGAS for recognition of dsDNA

**Fig. 5:**
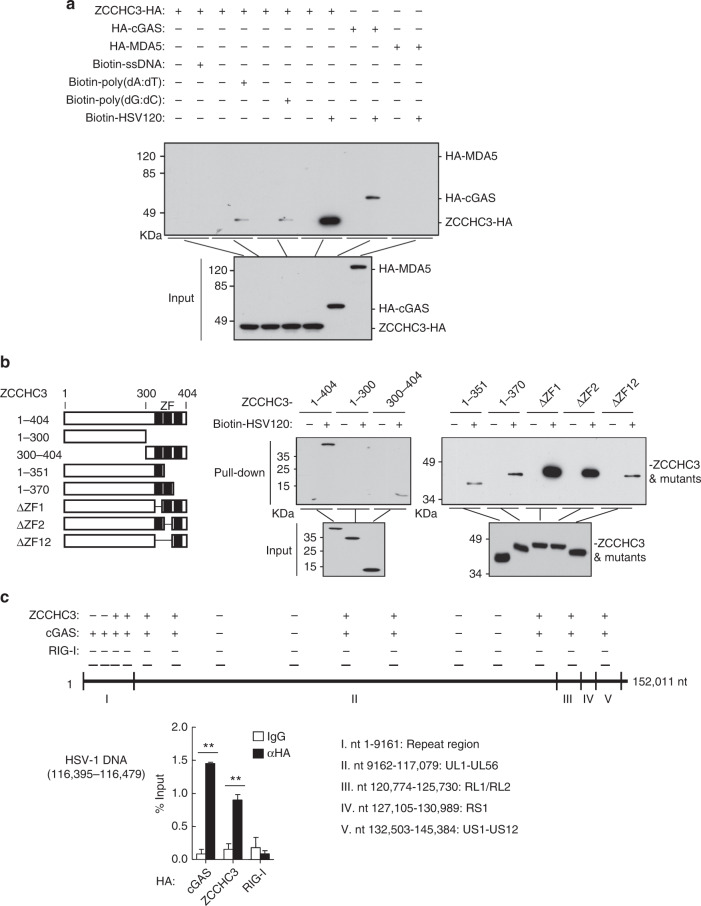
ZCCHC3 binds to dsDNA. **a** ZCCHC3 binds to dsDNA. HEK293 cells were transfected with the indicated plasmids. Twenty hours later, the cell lysates were incubated with the indicated biotinylated nucleic acids and streptavidin-Sepharose beads for in vitro pull-down assays. The bound proteins were then analyzed by immunoblots with anti-HA. **b** ZCCHC3 binds to dsDNA through its C-terminal ZF domains. HEK293 cells were transfected with the indicated plasmids. Twenty hours after transfection, the cell lysates were incubated with biotinylated-HSV120 and streptavidin-Sepharose beads. The bound proteins were analyzed by immunoblots with anti-Flag. A schematic representation of ZCCHC3 and its truncation mutants was shown on the left. **c** ZCCHC3 and cGAS but not RIG-I bind to HSV-1 DNA of infected cells. HEK293 cells were transfected with HA-tagged ZCCHC3, cGAS, and RIG-I. Twenty hours after transfection, cells were infected with HSV-1for 3 h. Cell lysates were then immunoprecipitated with control IgG or anti-HA. The protein-bound DNAs were extracted and analyzed by qPCR analysis with primers corresponding to the indicated regions of HSV-1 genome. Positive ( + ) and negative (-) detections were shown at the top of the schematic presentation of the HSV-1 genome. A representative qPCR results were shown at the left. **P < 0.01 (unpaired *t* test). Data are representative of three experiments with similar results

Since ZCCHC3 is a CCHC-type ZF protein which has been expected to bind to nucleic acids, we next examined whether ZCCHC3 binds to dsDNA. As shown in Fig. 5a, ZCCHC3 could bind to dsDNA such as poly(dA:dT) (B-DNA), poly(dG:dC) (Z-DNA) and HSV120 but not ssDNA in pull-down assays. In these experiments, the RNA sensor MDA5 did not bind to dsDNA. Deletion analysis indicated that the three ZFs could independently bind to HSV120 (Fig. 5b).

We further examined whether ZCCHC3 binds to viral DNA in infected cells by “foot-print” experiments. Following HSV-1 infection, we immunoprecipitated cGAS, ZCCHC3, and RIG-I, and the protein-bound viral DNA was detected by qPCR with primers targeting various regions of HSV-1 DNA. The results indicated that cGAS and ZCCHC3 but not RIG-I could bind to HSV-1 DNA, and in most cases to overlapping regions of the HSV-1 genome (Fig. 5c).

**Fig. 6:**
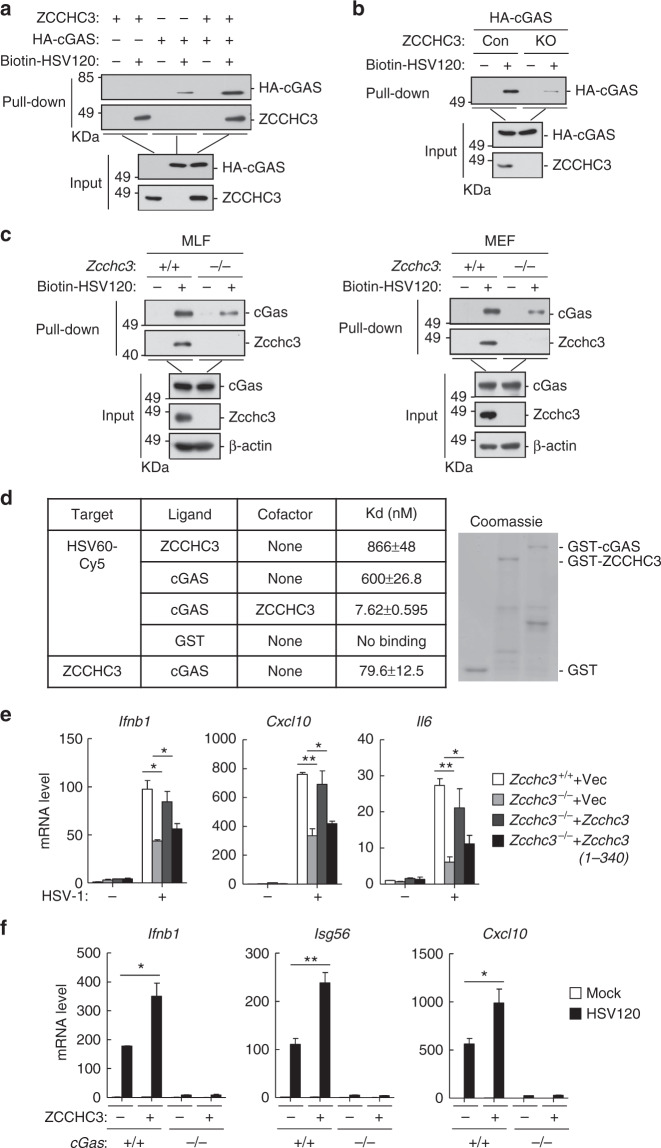
ZCCHC3 binds to dsDNA and facilitates the binding of cGAS to dsDNA. **a** ZCCHC3 enhances the binding of cGAS to dsDNA. HEK293 cells were transfected with the indicated plasmids. Twenty hours later, the cell lysates were incubated with biotinylated-HSV120 and streptavidin-Sepharose beads for in vitro pull-down assays. The bound proteins were then analyzed by immunoblots with anti-HA and anti-ZCCHC3. **b** ZCCHC3-deficiency decreases the binding of cGAS to dsDNA in HEK293 cells. ZCCHC3-KO HEK293 clones were generated by the CRISPR-Cas9 method. ZCCHC3-KO and control HEK293 cells were transfected with HA-cGAS. Twenty hours after transfection, cells were collected for in vitro pull-down assays similarly as in **a**. **c** ZCCHC3-deficiency decreases the binding of endogenous cGAS to dsDNA in MLFs or MEFs. *Zcchc3*^*+/+*^ and *Zcchc3*^−*/*−^ MLFs or MEFs were collected for in vitro pull-down assays. The bound proteins were then analyzed by immunoblots with anti-cGAS and anti-ZCCHC3. **d** MST measurement of binding affinities. Binding affinities between the indicated recombinant proteins as well as between the indicated recombinant proteins and synthetic dsDNA HSV60 were measured by MST. The purified recombinant proteins were stained with Coomassie blue (right gel). **e** Effects of reconstitution of ZCCHC3 or ZCCHC3 (1-340) on HSV-1-induced transcription of downstream genes in *Zcchc3*^−*/*−^MLFs. *Zcchc3*^−*/*−^ MLFs were reconstituted with murine ZCCHC3 or ZCCHC3(1-340) by lentiviral-mediated gene transfer. The reconstituted MLFs were left un-infected or infected with HSV-1 for 6 h before qPCR analysis. **f** Effects of ZCCHC3 on transcription of downstream genes induced by HSV120 in *cGas*^*+/+*^ and *cGas*^−*/*−^ L929 cells. *cGas*^*+/+*^ and *cGas*^−*/*−^ L929 cells stably expressing ZCCHC3 were transfected with HSV120 (3 μg/ml) for 4 h before qPCR analysis. *P < 0.05, **P < 0.01 (unpaired *t* test). Data are representative of at least two experiments with similar results (mean ± SD, *n* = 3 independent samples in **e**, **f**)

We further investigated whether and how ZCCHC3 modulates cGAS activity. In vitro pull-down analysis indicated that overexpression of ZCCHC3 enhanced the binding of cGAS to HSV120 (Fig. 6a), whereas ZCCHC3-deficiency inhibited the binding of cGAS to HSV120 in HEK293 cells (Fig. 6b). Consistently, ZCCHC3-deficiency inhibited the binding of endogenous cGAS to HSV120 in primary MLFs and MEFs (Fig. 6c). We have further produced recombinant ZCCHC3 and cGAS, and then examined the binding affinities of these proteins to dsDNA by Microscale thermophoresis technology (MST). The results indicated that ZCCHC3 and cGAS could bind to the synthetic dsDNA HSV60 with an affinity of Kd = 866±48 or 600±26.8 nM respectively. In addition, recombinant ZCCHC3 bound to cGAS with a higher affinity (Kd = 79.6±12.5 nM). The complex of ZCCHC3 and cGAS could bind to HSV60 with a dramatic higher affinity (Kd = 7.62±0.595 nM) than each of them alone (Fig. 6d). Collectively, these results suggest that ZCCHC3 promotes recognition of dsDNA by cGAS.

We further investigated whether ZCCHC3 promotes sensing of dsDNA by cGAS is dependent on its DNA binding capacity. As shown in Fig. 6e, reconstitution of ZCCHC3 but not its mutant lacking the ZF domains rescued HSV-1-induced transcription of *Ifnb1*, *Cxcl10*, and *Il6* genes in *Zcchc3*^−*/*−^ MLFs, suggesting that the DNA binding capacity of ZCCHC3 is important for its role in modulating cGAS activation.

Since ZCCHC3-deficiency causes marked but not complete inhibition of dsDNA-induced signaling, whereas cGAS-deficiency has a more dramatic effect, we determined whether the effects of ZCCHC3 on dsDNA-induced signaling are mediated by cGAS. qPCR analysis indicated that overexpression of ZCCHC3 potentiated transcription of *Ifnb1*, *Isg56*, and *Cxcl10* genes following HSV120 transfection in wild-type L929 cells. However, cGAS-deficiency dramatically inhibited HSV120-induced and ZCCHC3-mediated transcription of the downstream genes (Fig. 6f), suggesting that the effects of ZCCHC3 on dsDNA-induced transcription of downstream genes are mediated by cGAS.

We further investigated whether the expression of endogenous ZCCHC3 and cGAS in various cells and tissues is correlated. We found that ZCCHC3 was ubiquitously expressed in different human cell lines including THP1, HFF, HCT116, A549, HepG2, and HeLa cells, as well as primary human immune cells including macrophages and DCs (Supplementary Fig. 4A). The expression levels of ZCCHC3 were correlated to those of cGAS in all the examined human cell lines or primary immune cells except HCT116 (Supplementary Fig. 4A). We also found that the mRNAs of both Zcchc3 and cGas were expressed widely in most mouse tissues (Supplementary Fig. 4B). Transcription of *cGas* but not *Zcchc3* was induced by viral infection or IFN-β stimulation in murine lung fibroblasts (MLFs) (Supplementary Fig. 4C).

### ZCCHC3 modulates cGAS oligomerization

**Fig. 7:**
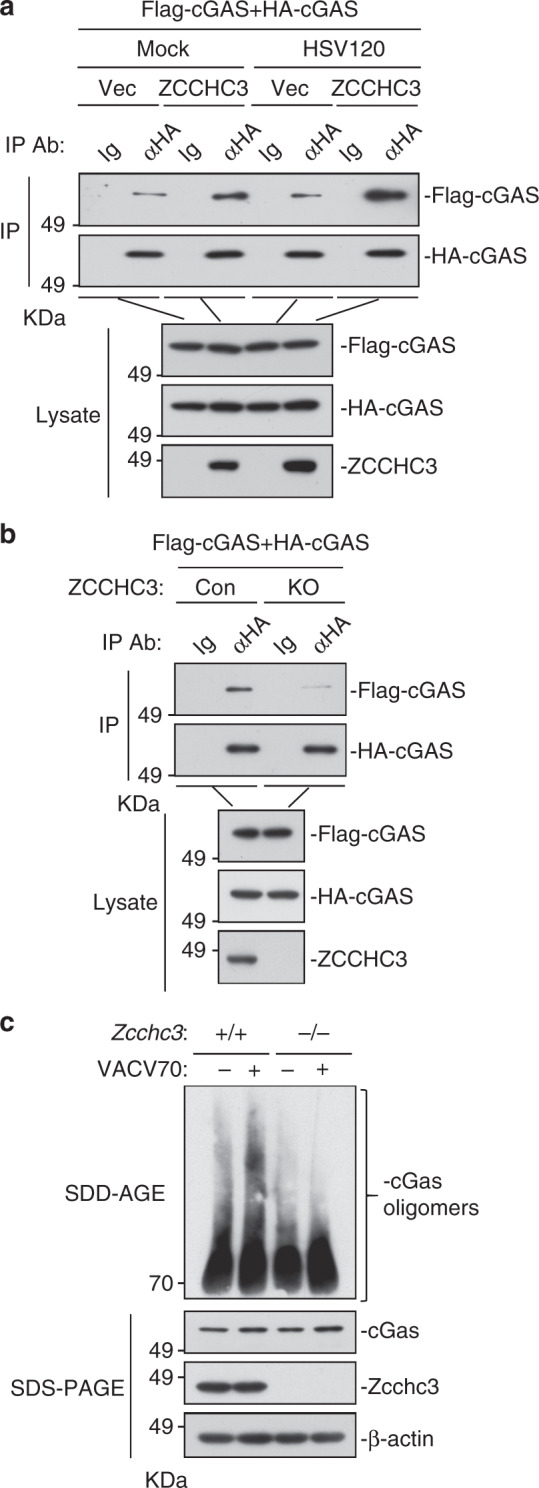
ZCCHC3 is important for cytosolic dsDNA-induced oligomerization of cGAS. **a** Effects of ZCCHC3 on self-association of cGAS. HEK293 cells were transfected with the indicated plasmids for 20 h. The cells were then left un-treated or treated with HSV120 for 4 h before co-immunoprecipitation and immunoblotting analysis with the indicated antibodies. **b** Effects of ZCCHC3-deficiency on self-association of cGAS. ZCCHC3-KO and control HEK293 cells were transfected with the indicated plasmids for 20 h before co-immunoprecipitation and immunoblotting analysis with the indicated antibodies. **c** Effects of ZCCHC3-deficiency on oligomerization of cGAS induced by transfected VACV70. *Zcchc3*^*+/+*^ and *Zcchc3*^−*/*−^ MLFs were left un-treated or treated with VACV70 for 4 h. Cell lysates were then fractionated by SDD-AGE and SDS-PAGE and analyzed by immunoblots with the indicated antibodies. Data are representative of three experiments with similar results

Previously, it has been shown that cGAS oligomerization is important for its activation after binding to dsDNA^34^. Co-immunoprecipitation experiments indicated that ZCCHC3 enhanced self-association of cGAS, which was further enhanced following stimulation with transfected HSV120 (Fig. 7a). Conversely, ZCCHC3-deficiency markedly inhibited the self-association of cGAS in HEK293 cells (Fig. 7b). In addition, oligomerization of cGAS induced by transfected VACV70 was decreased in *Zcchc3*^−*/*−^ in comparison to *Zcchc3*^*+/+*^ MLFs (Fig. 7c).

In our earlier experiments (Fig. 4a), we have shown that ZCCHC3-deficiency dramatically inhibited cytosolic dsDNA-triggered synthesis of cGAMP (Fig. 4a). Collectively, these results suggest that ZCCHC3 is important for cytosolic dsDNA-induced oligomerization and enzymatic activity of cGAS.

## Discussion

It has been well established that cGAS is a ubiquitous sensor for cytosolic dsDNA, which plays an essential role in innate immune response to microbial and dis-located self DNA^15,23^. Since cGAS recognizes dsDNA with low specificity, it is unknown how cGAS acts as an efficient and specific sensor for cytosolic dsDNA. In this study, we identified the CCHC-type ZF protein ZCCHC3 as a co-sensor for cGAS, which is required for efficient innate immune response to cytosolic and viral dsDNA.

Several lines of evidence suggest that ZCCHC3 plays an important role in innate immune response to cytosolic dsDNA and DNA virus. Overexpression of ZCCHC3 potentiated HSV-1- or cytosolic dsDNA-induced transcription of downstream effector genes, whereas knockout of ZCCHC3 had the opposite effects in various cell types. Reconstitution of ZCCHC3 rescued HSV-1-triggered induction of downstream cytokines in *Zcchc3*^−*/*−^ cells. The serum cytokines such as IFN-β, CXCL10, and IL-6 induced by infection of HSV-1, VACV, and MCMV were impaired in *Zcchc3*^−*/*−^ in comparison to *Zcchc3*^*+/+*^ mice, and ZCCHC3-deficiency renders the mice more susceptible to HSV-1- or VACV-induced death. The amounts of HSV-1 genomic copy numbers and viral titers were markedly increased in the brains of *Zcchc3*^−*/*−^ mice in comparison to their wild-type counterparts after HSV-1 infection. In addition to a role in innate immune response to DNA virus, we also found that ZCCHC3 plays an important role in innate immune response to RNA virus, which will be characterized and reported in a separate study.

Mechanistic studies suggest that ZCCHC3 acts as a general co-sensor for recognition of dsDNA by cGAS. Firstly, ZCCHC3-deficiency inhibited HSV-1-triggered induction of cGAMP but not cGAMP-induced transcription of downstream effector genes. In addition, ZCCHC3-mediated induction of downstream genes was abolished in *cGas*^−*/*−^ cells. Cellular and biochemical experiments indicated that ZCCHC3 interacted with cGAS, and their endogenous association was induced by HSV-1 infection or dsDNA stimulation. These results suggest that ZCCHC3 functions through cGAS. Second, in vitro pull-down analysis showed that ZCCHC3 itself bound to dsDNA through its ZF domains. cGAS also contains a CCHC-type ZF motif that is required for its binding to dsDNA^15,33,40^. Foot-print experiments indicated that ZCCHC3 and cGAS bound to overlapping regions of naturally infected HSV-1 DNA. In mammalian cells, overexpression of ZCCHC3 promoted the binding of cGAS to dsDNA, whereas ZCCHC3-deficiency had opposite effects. Third, MST experiments with purified recombinant proteins indicated that ZCCHC3 and cGAS could bind to synthetic dsDNA with similar affinity. ZCCHC3 and cGAS could also bind to each other with a higher affinity than their respective affinities to dsDNA. The complex of ZCCHC3 and cGAS could bind to dsDNA with a dramatic higher affinity than each of them alone. Collectively, these results suggest that ZCCHC3 acts as a co-sensor of cGAS to promote its binding to dsDNA and subsequent enzymatic activation.

Based on our results, we conclude that viral infection induces the association of ZCCHC3 and cGAS, which have a high affinity to viral dsDNA, leading to cGAS activation and induction of downstream effector genes. Currently, we do not know whether individual binding of ZCCHC3 or cGAS to dsDNA promotes the interaction of the two proteins, and subsequently increases the affinity of the complex to dsDNA, or alternatively, viral infection firstly induces formation of ZCCHC3 and cGAS complex through unknown mechanisms, which has much higher affinity to dsDNA than each of them alone. It has been shown that the cellular protein PQBP1 serves as a co-sensor for recognition of reverse-transcribed HIV-1 ssDNA by cGAS, but is not required for cGAS-dependent responses to cytosolic dsDNAs^38^. In light of this observation, ZCCHC3 may act as a general co-sensor of cGAS for recognition of cytosolic dsDNA originated from dis-located self or microbial DNA, and the identification of ZCCHC3 represents an important step towards our understanding of innate immune response to dis-located self DNA or DNA pathogens.

## Methods

### Mice

*Zcchc3*^−*/*−^ mice on the C57BL/6 background were generated by the CRISPR/Cas9 method and obtained from the Wuhan University A3 Animal Center. The mice were bred in specific pathogen-free facilities at Wuhan University College of Life Sciences. The animal care and use protocol was adhered to the Chinese National Laboratory Animal-Guideline for Ethical Review of Animal Welfare. The protocols and procedures for mice experiments in this study were approved by the Wuhan University College of Life Sciences Animal Care and Use Committee (approval number WDSKY0200902-2). Six to eight week-old mice were used in the experiments and littermates were used as controls. Experiments were conducted without blinding, with age- and sex-matched mice.

### Reagents, antibodies, cells, and viruses

Poly(dA:dT), 2′ 3′-cGAMP, and Lipofectamine 2000 (InvivoGen); polybrene (Millipore); SYBR (Bio-Rad); FuGene and Dual-Specific Luciferase Assay Kit (Promega); digitonin (Sigma); puromycin and EZ-link Psoralen-PEG_3_-Biotin (Thermo); streptavidin agarose (Solulink); ELISA kits for murine IFN-β (PBL), IL-6 (BioLegend), and CXCL10 (BOSTER); recombinant IFN-β (R&D systems) were purchased for the indicated manufacturers.

Mouse monoclonal antibodies against HA (BioLegend, 901515; 1:2000), FLAG (Sigma, F3165; 1:2000) and β-actin (Sigma, A2228; 1:3000) and phosphor-IκBα (Cell Signaling Technology, 9246 S; 1:1000); rabbit monoclonal antibodies against cGAS (Cell Signaling Technology, 66546 S/31659 S; 1:1000), phosphor-Tyrosine701-STAT1 (Cell Signaling Technology, 9167 S; 1:1000) and phosphor-IRF3 (Cell Signaling Technology, 4947 S; 1:1000), phosphor-TBK1 (Abcam, ab109272; 1:1000) and TBK1(Abcam, ab40676; 1:2000), IRF3 (Santa Cruz Biotechnology, sc-33641; 1:1000) and STAT1 (Santa Cruz Biotechnology, sc-417; 1:1000) were purchased from the indicated manufacturers. Antisera against ZCCHC3 were generated by immunizing rabbits or mice with purified recombinant ZCCHC3(133-404) (1:1000).

HEK293 cells (Cat # CRL-11268) and THP1 cells (Cat # TIB-202) were obtained from ATCC. HFFs were provided by Dr. Min-Hua Luo (Wuhan Institute of Virology, CAS). HSV-1 (KOS strain), VACV (Tian-Tan Strain) and MCMV were purchased from China Center for Type Culture Collection.

### Constructs

Expression plasmids for HA-, FLAG-, or GFP-tagged ZCCHC3 and its truncation mutants, and HA-, FLAG-, or RFP-tagged cGAS and its truncation mutants were constructed by standard molecular biology techniques. Expression plasmids for HA-tagged MDA5, MITA, TBK1, and IRF3, and the IFN-β promoter reporter plasmids were previously described^30,41–44^.

### Preparations of primary mouse cells

For preparation of BMDMs, mouse bone marrow-derived monocytes (5 × 10^6^) were cultured in 100-mm dishes in 5 ml 10% M-CSF-containing conditional medium from L929 cells for 3–5 days. For preparation of BMDCs, mouse bone marrow-derived monocytes (5 × 10^6^) were cultured in medium containing murine GM-CSF (50 ng/ml) for 6–8 days. For preparation of lung fibroblasts, lungs were minced and digested in calcium and magnesium free HBSS containing 10 μg/ml type II collagenase and 20 μg/ml DNase I for 1 h at 37 °C with shaking. Cell suspension was centrifuged at 448 × *g* for 5 min. The cells were then plated in culture medium (1:1 [v/v] DMEM/Ham’s F-12 containing 10% FBS, 50 U/ml penicillin, 50 μg/ml streptomycin, 15 mM HEPES, 2 mM l-glutamine). MEFs were prepared from day 12.5 embryos and cultured in DMEM supplemented with 10% FBS.

### DNA oligonucleotides

The following oligonucleotides were used to stimulate cells:

ISD45: 5’-TACAGATCTACTAGTGATCTATGACTGATCTGTACATGATCTACA-3’;

dsDNA90: 5’-TACAGATCTACTAGTGATCTATGACTGATCTGTACATGATCTACATACAGATCTACTAGTGATCTATGACTGATCTGTACATGATCTACA-3’;

VACV70: 5’-CCATCAGAAAGAGGTTTAATATTTTTGTGAGACCATGGAAGAGAGAAAGAGATAAAACTTTTTTACGACT-3’;

HSV120: 5’-AGACGGTATATTTTTGCGTTATCACTGTCCCGGATTGGACACGGTCTTGTGGGATAGGCATGCCCAGAAGGCATATTGGGTTAACCCCTTTTTATTTGTGGCGGGTTTTTTGGAGGACTT-3’.

### Transfection and reporter assays

HEK293 cells were transfected by standard calcium phosphate precipitation method. HeLa cells were transfected by FuGENE. HFFs, MLFs, and MEFs were transfected by Lipofectamine 2000. To ensure that each transfection receives the same amount of total DNA, the empty control plasmid was added to each transfection. To normalize for transfection efficiency, pRL-TK (*Renilla* luciferase) reporter plasmid (0.01 μg) was added to each transfection. Luciferase assays were performed using a Dual-Specific Luciferase Assay Kit. Firefly luciferase activities were normalized on the basis of *Renilla* luciferase activities.

### qPCR

Total RNA was isolated for qPCR analysis to measure mRNA abundance of the indicated genes. Data shown are the relative abundance of the indicated mRNA derived from human or mouse cells normalized to that of GAPDH. Gene-specific primer sequences were listed in Supplementary Table 1.

### CRISPR-Cas9 knockout

Genome engineering was performed using the CRISPR-Cas9 system^45,46^. Double-stranded oligonucleotides corresponding to the target sequences were cloned into the lenti-CRISPR-V2 vector and cotransfected with packaging plasmids into HEK293 cells. Two days after transfection, the viruses were harvested and used to infect HFFs or THP1 cells. The infected cells were selected with puromycin (1 μg/ml) for at least 5 days. The following sequences were targeted for human ZCCHC3 cDNA. #1: 5′-AGGGCGAATTCCGCGAGCCG-3′; and #2: 5′-CGGCCGCGAGAAGATGGGCT-3′.

### Viral plaque assays

Eight-week-old mice were infected with HSV-1 for 6–7 days, the brains of mice were weighed and homogenized for 5 s in PBS. After homogenization, the brain suspensions were centrifuged at 1620 × *g* for 30 min, and the supernatants were used for plaque assays. Vero cells were seeded in 24-well plates, and the cells were infected by incubation for 2 h at 37 °C with serial dilutions of the brain suspensions. After infection for 2 h, 2% methylcellulose was overlaid, and the plates were incubated for 36–48 h. The overlay was removed, and cells were fixed with 4% paraformaldehyde for 20 min and stained with 1% crystal violet for 20 min before plaque counting.

### ELISA

BMDCs were stimulated with viruses or transfected with synthetic nucleic acids for 18 h. The culture media were collected for measurement of IFN-β and IL-6. Eight-week-old *Zcchc3*^*+/+*^ and *Zcchc3*^−*/*−^ mice were intra-nasally infected with HSV-1, VACV, and MCMV for 6 h, and then the sera of mice were collected for measurement of IFN-β, CXCL10, and IL-6 by ELISA.

### Co-immunoprecipitation and immunoblotting analysis

Cells were lysed in NP-40 lysis buffer (20 mM Tris-HCl pH 7.4, 150 mM NaCl, 1 mM EDTA, 1% NP-40, 10 μg/ml aprotinin, 10 μg/ml leupeptin, 1 mM phenylmethylsulfonyl fluoride). For each immunoprecipitation, a 0.4-mL aliquot of the lysate was incubated with the indicated antibody or control IgG (0.5 μg, or 0.5 μl for antiserum) and 25 μL of a 1:1 slurry of Protein G Sepharose (GE Healthcare) for 2 h. Sepharose beads were washed three times with 1 mL of lysis buffer containing 0.5 M NaCl. The precipitates were analyzed by standard immunoblot procedures. Uncropped scans of the immunoblots are provided in Supplementary Figs. 5–12.

### In vitro pull-down assays

HEK293 cells transfected with the indicated plasmids were lysed in NP-40 lysis buffer. Lysates were incubated with biotinylated-HSV120 for 1 h at 4 °C, and then incubated with streptavidin beads for another 2 h at 4 °C. The beads were washed three times with lysis buffer and analyzed by immunoblotting with the indicated antibodies.

### Fluorescent confocol microscopy

HeLa cells were transfected with the indicated plasmids by FuGENE. After transfection for 20 h, the cells were fixed with 4% paraformaldehyde for 15–20 min and then washed with PBS for 3 times. The nuclei were stained with DAPI for 2 min and then washed with PBS for 3 times. Imaging of the cells was carried out using Nikon A1 MP confocal microscope under a ×60 oil objective.

### Proximity ligation assay (PLA)

The PLA reagents were purchased from Sigma and the experiments were performed following the manufacturer’s instructions. Briefly, MEFs were seeded on Teflon-coated glasses and cultured for 18 h. After HSV-1 infection or transfection of HSV120 for 4 h, cells were fixed with 4% paraformaldehyde for 15–20 min at room temperature followed by permealization with 0.1% TritonX-100 for 10 min. Cells were blocked in 5% BSA in PBS for 30 min and incubated in primary antibodies for 2 h. Cells were washed with wash buffer A (DUO82049) for 3 times and incubated with the secondary antibodies with PLA probes (DUO92004-30RXN, DUO92002-30RXN) for 2 h at 37 °C followed by 3 times wash with the wash buffer A. The cells were then incubated with the Ligation-Ligase solution (DUO92008) for 30 min at 37 °C followed by 3 times wash with the wash buffer A. Cells were incubated with Amplification-Polymerase solution (DUO92008) for 100 min at 37 °C followed by 3 times wash with the wash buffer B (DUO82049). The nuclei were stained with DAPI for 2 min and then washed with PBS for 3 times. Imaging of the cells was carried out using Nikon A1 MP confocal microscope under a ×60 oil objective.

### Recombinant proteins purification

The cDNAs encoding for ZCCHC3 and cGAS were cloned into the pGEX-6p-1-GST plasmid. The plasmids were transformed into the BL21 E. Coli strain. Expression of the GST-fusion proteins were induced with 0.1 mM IPTG at 16 °C for 24 h. The recombinant proteins were purified with GST resins and eluted with elution buffer (PBS, 100 mM Tris-HCl pH 8.8, 40 mM reduced glutathione).

### Microscale thermophoresis technology (MST)

MST analysis was performed using a NanoTemper Monolith NT.115 instrument (NanoTemper Technologies GmbH). For detecting affinity between GST-ZCCHC3 or GST-cGAS and dsDNA, 20 nM Cy5-labeled 60 bp dsDNA HSV60 (Sangon Biotech, China) was mixed with different concentrations of proteins in PBS with 100 mM Tris-HCl, pH 8.8. For detecting affinity between GST-ZCCHC3 and GST-cGAS, GST-ZCCHC3 was fluorescently labeled with a Blue-NHS labeling kit according to the manufacturer’s instructions. Lysine residues of GST-ZCCHC3 were labeled with a dye: protein molar ratio of 3. For detecting affinity between cGAS/ZCCHC3 complex and HSV60, 20 nM Cy5-labeled HSV60 was mixed with different concentrations of GST-cGAS saturated with GST-ZCCHC3 dependent on their Kd values. In these experiments, GST was used as negative control. Samples were loaded into Premium Coated Capillaries and MST measurements were performed using 20% MST power and 40% LED power at 25 °C. Laser-on and -off times were 30 and 5 s respectively. NanoTemper Analysis 1.2.20 software was used to fit the data and to determine the apparent Kd values.

### Immuno-precipitation for viral DNA (Foot-print assay)

HEK293 cells were transfected with HA-tagged ZCCHC3, cGAS and RIG-I for 20 h. The cells were then infected with HSV-1 for 1 h, washed with medium and cultured for 2 more hours. Cell lysates were immunoprecipitated with IgG or anti-HA (2 μg) and protein G beads (50 μl) at 4 °C for 4 h. Before DNA extraction, a fraction of agarose beads were eluted using 2xSDS loading buffer, and boiled for 10 min by SDS-PAGE to confirm the protein precipitation. The bead-bound immunoprecipitates were washed for 3 times with lysis buffer containing 1 M NaCl. The beads were treated with elution buffer (0.1 M NaHCO3, 1% SDS) containing protease K for two times, and the supernatants were incubated for 30 min at 65 °C and subjected to phenol-chlorophorm extraction for 4 times. The aqueous phase was collected and mixed with ethanol, NaOAc and glycogen and store at −20 °C for at least 30 min. DNAs were precipitated by centrifugation for 30 min at 4 °C, washed with 75% ethanol and air-dried before resuspension in ddH_2_O for qPCR analysis of HSV-1 DNA. The following sequence is targeted for HSV-1 genome. nt 116395-116479: 5′-ACGACAGTGGCATAGGTTGG-3′ (forward), 5′-CCGACATCACAAGGGACCTC-3′ (reverse).

### Semi-denaturing detergent agarose gel electrophoresis (SDD-AGE)

MLFs were lysed in NP-40 lysis buffer, and the cell lysates were mixed in 1x sample buffer (0.5xTBE, 10% glycerol, 2% SDS, and 0.0025% bromophenol blue) and loaded onto a vertical 2% agarose gel (Bio-Rad). After electrophoresis in the running buffer (1xTBE and 0.1% SDS) for about 2 h with a constant voltage of 100 V at 4 °C, the proteins were transferred to immobilon membrane (Millipore) for immunoblotting analysis.

### Digitonin permeabilization

Cells were mock-transfected or transfected with HSV120 (3 μg/ml) for 4 h. Cell extracts were then prepared and heated at 95 °C for 5 min to denature most proteins, which were removed by centrifugation. The supernatants containing cGAMP were delivered to MLFs pretreated with digitonin permeabilization solution (50 mM HEPES pH 7.0, 100 mM KCl, 3 mM MgCl_2_, 0.1 mM DTT, 85 mM Sucrose, 0.2% BSA, 1 mM ATP, 0.1 mM GTP and 10 μg/ml digitonin) at 37 °C for 30 min. Three hours later, the cells were collected for a qPCR analysis.

### Statistics

Unpaired Student’s *t* test was used for statistical analysis with GraphPad Prism Software. For the mouse survival study, Kaplan–Meier survival curves were generated and analyzed by Log-Rank test; *P* < 0.05 was considered significant.

### Data availability

The data that support the findings of this study are available from the corresponding authors upon request.

## Electronic supplementary material

Supplementary Information
