# Author Correction: ZCCHC3 is a co-sensor of cGAS for dsDNA recognition in innate immune response

**DOI:** 10.1038/s41467-021-25397-7

**Published:** 2021-09-14

**Authors:** Huan Lian, Jin Wei, Ru Zang, Wen Ye, Qing Yang, Xia-Nan Zhang, Yun-Da Chen, Yu-Zhi Fu, Ming-Ming Hu, Cao-Qi Lei, Wei-Wei Luo, Shu Li, Hong-Bing Shu

**Affiliations:** 1 Medical Research Institute, School of Medicine, Wuhan University, 430071 Wuhan, China; 2 College of Life Sciences Wuhan University, 430072 Wuhan, China; 3 Wuhan Institute of Virology, Chinese Academy of Sciences, 430071 Wuhan, China

**Keywords:** Antimicrobial responses, Innate immunity, Viral host response, Infection

Correction to: *Nature Communications* 10.1038/s41467-018-05559-w, published online 22 August 2018.

The original version of this Article contained errors in the listed sequences for GADPH, ISG56, IL-6 and RIG-I in Supplementary Table 1. The labelled orientations of primer sequences for GADPH were also incorrect. The Rantes primers were incorrectly included in the original Supplementary Table 1.

The forward primer sequence for GADPH has been corrected from ‘GAGTCAACGGATTTGGTGGT’ to ‘GAGTCAACGGATTTGGTCGT’. ‘GACAAGCTTCCCGTTCTCAG’ is now correctly listed as the reverse primer for GADPH and ‘GAGTCAACGGATTTGGTCGT’ as the forward primer.

The ISG56 reverse primer has been corrected from ‘CCACACTGTATTTGGTGTCTACG’ to ‘CCACACTGTATTTGGTGTCTAGG’.

The forward and reverse primer sequences for IL-6 have been corrected from ‘TCTGCAAGAGACTTCCATCCAGTTGC‘ to ‘TTCTCCACAAGCGCCTTCGGTC’ and ‘AGCCTCCGACTTGTGAAGTGGT’ to ‘TCTGTGTGGGGCGGCTACATCT’, respectively.

The forward and reverse primer sequences primer sequences for RIG-I have been corrected from ‘GAGGAGGTGAAAGACCAGAGCA’ to ‘ACGCAGCCTGCAAGCCTTCC’ and ‘TAGCATCTCGGCTGGACTTCGA’ to ‘TGTGGCAGCCTCCATTGGGC’, respectively.

The Rantes primers have been deleted from Supplementary Table 1.

The HTML has been updated to include a corrected version of the Supplementary Information. The correct version of the Supplementary Information can be found as Supplementary Information associated with this correction.

## Supplementary information

Supplementary Information

