# Supplementary material for: ZCCHC3 is a co-sensor of cGAS for dsDNA recognition in innate immune response
Source: Nat Commun. 2018 Aug 22;9:3349. doi: 10.1038/s41467-018-05559-w (PMC6105683; doi:10.1038/s41467-018-05559-w)
Supplement: Supplementary file 1 — Supplementary Information [file 41467_2018_5559_MOESM1_ESM.pdf]

Supplementary Information

ZCCHC3 is a co-sensor of cGAS for dsDNA recognition in innate immune response

Lian et al.

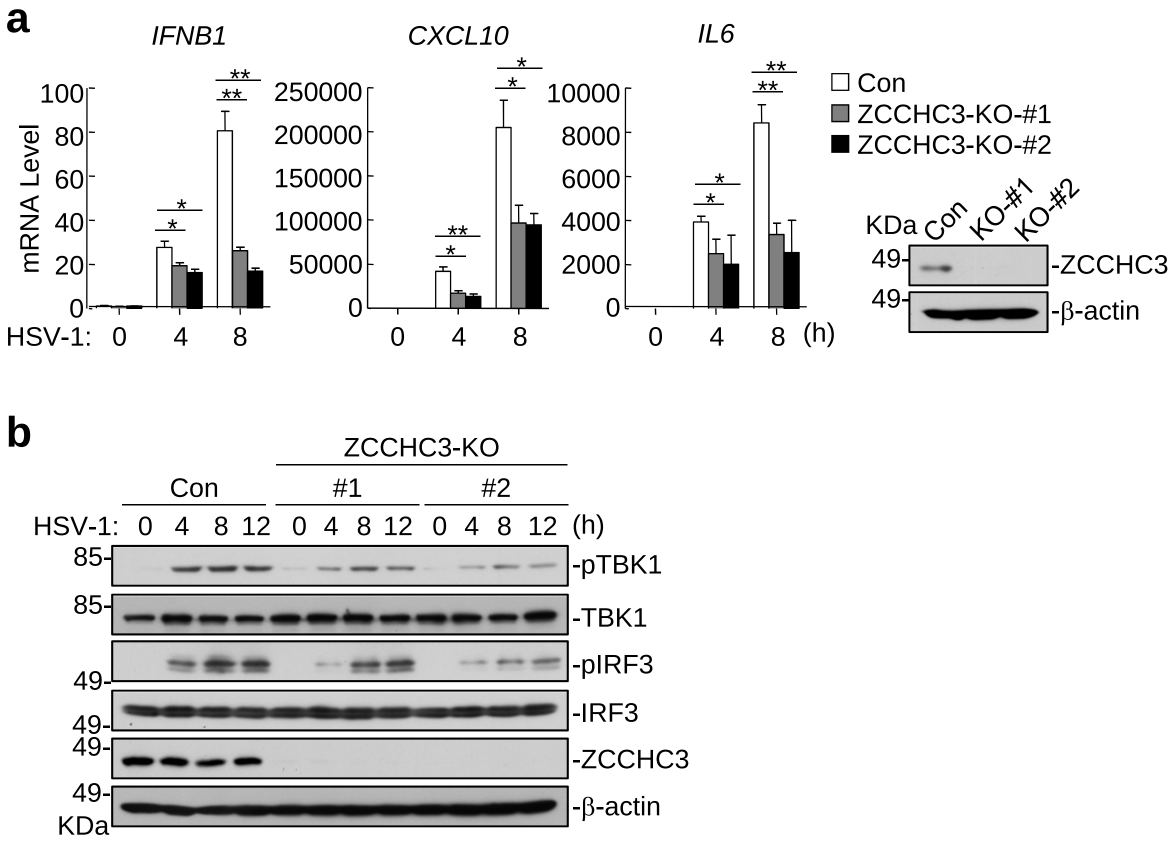

Supplementary Figure 1. ZCCHC3-deficiency inhibits HSV-1-induced signaling in THP1 cells.
a Effects of ZCCHC3-deficiency on HSV-1-induced transcription of downstream genes. ZCCHC3-KO and control THP1 cells were left un-infected or infected with HSV-1 for the indicated times before qPCR analysis. ZCCHC3-deficiency in the KO THP1 cells was confirmed by immunoblotting analysis (right blots). * $P < 0.05$, ** $P < 0.01$ (unpaired *t* test). Data are representative of three experiments with similar results (mean ± SD, n=3 independent samples). **b** Effects of ZCCHC3-deficiency on HSV-1-induced phosphorylation of TBK1 and IRF3. ZCCHC3-KO and control THP1 cells were left un-infected or infected with HSV-1for the indicated times before immunoblotting analysis.

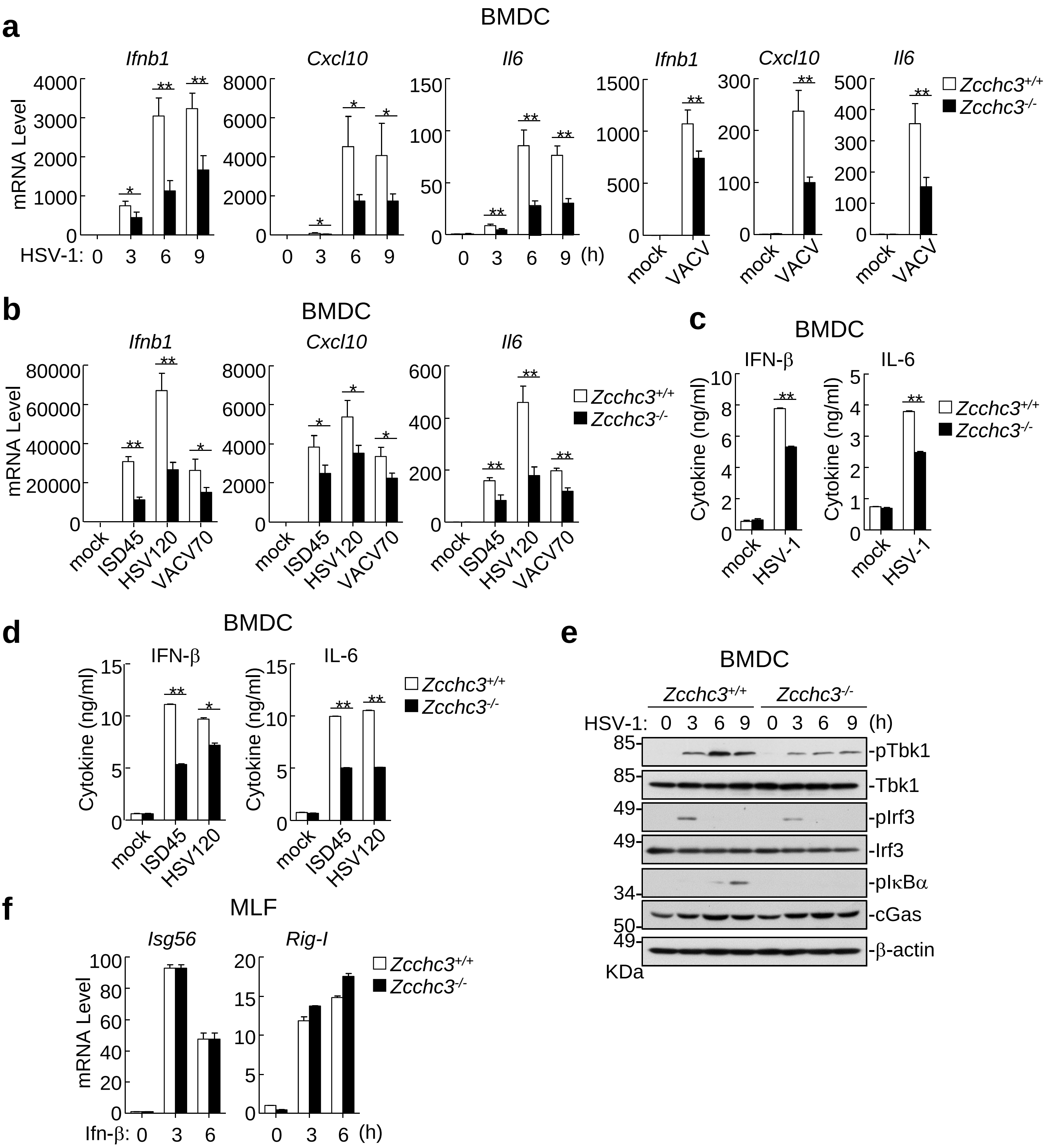

Supplementary Figure 2. Effects of ZCCHC3-deficiency on viral DNA-triggered signaling in murine cells.
a Effects of ZCCHC3-deficiency on DNA virus-induced transcription of downstream genes. *Zcchc3*$^{+/+}$ and *Zcchc3*$^{-/-}$ BMDCs were left un-infected or infected with HSV-1 for the indicated times or VACV for 6 h before qPCR analysis. **b** Effects of ZCCHC3-deficiency on dsDNA-induced transcription of downstream genes. *Zcchc3*$^{+/+}$ and *Zcchc3*$^{-/-}$ BMDCs were transfected with the indicated nucleic acids for 4 h before qPCR analysis. **c** Effects of ZCCHC3-deficiency on HSV-1-induced secretion of IFN-β and IL-6. *Zcchc3*$^{+/+}$ and *Zcchc3*$^{-/-}$ BMDCs were left un-infected or infected with HSV-1 for 18 h before ELISA for cytokine levels in the culture. **d** Effects of ZCCHC3-deficiency on dsDNA-induced secretion of of IFN-β and IL-6. *Zcchc3*$^{+/+}$ and *Zcchc3*$^{-/-}$ BMDCs were transfected with the indicated nucleic acids for 18 h before ELISA analysis similarly as in **c**. **e** Effects of ZCCHC3-deficiency on HSV-1-induced phosphorylation of TBK1, IRF3, and IκBα. *Zcchc3*$^{+/+}$ and *Zcchc3*$^{-/-}$ BMDCs were left un-infected or infected with HSV-1 for the indicated times before immunoblotting analysis. **f** Effects of ZCCHC3-deficiency on IFN-β-induced transcription of downstream genes in MLFs. *Zcchc3*$^{+/+}$ and *Zcchc3*$^{-/-}$ MLFs were left un-treated or treated with IFN-β for 6 h before qPCR analysis. * $P < 0.05$, ** $P < 0.01$ (unpaired *t* test). Data are representative of at least two experiments with similar results (mean ± SD, n=3 independent samples in **a-d**, **f**).

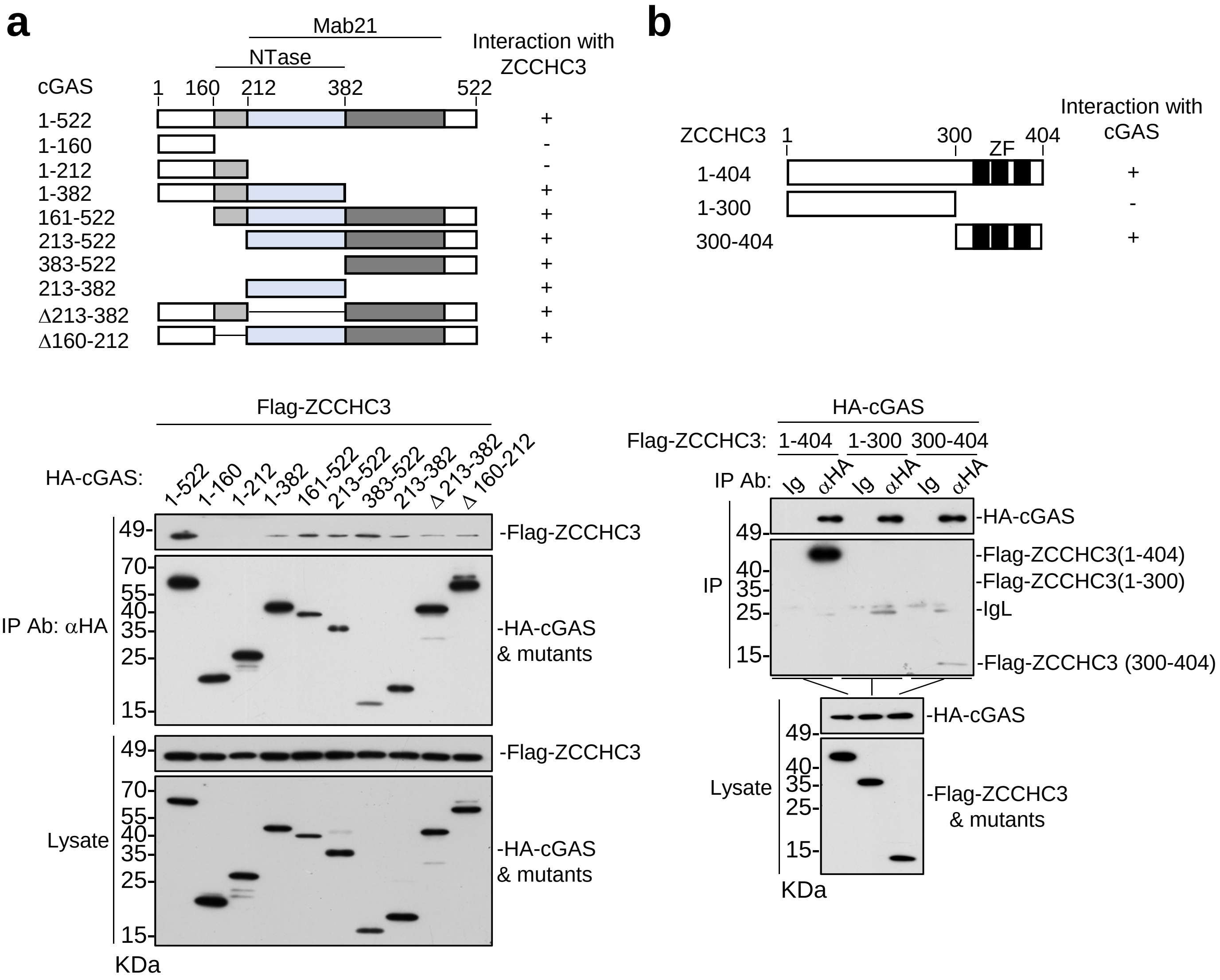

Supplementary Figure 3. Domain mapping of ZCCHC3-cGAS interaction.
HEK293 cells were transfected with the indicated truncations before co-immunoprecipitation and immunoblotting analysis with the indicated antibodies. The schematic presentations of cGAS and ZCCHC3 truncations are shown at the top. Data are representative of two experiments with similar results.

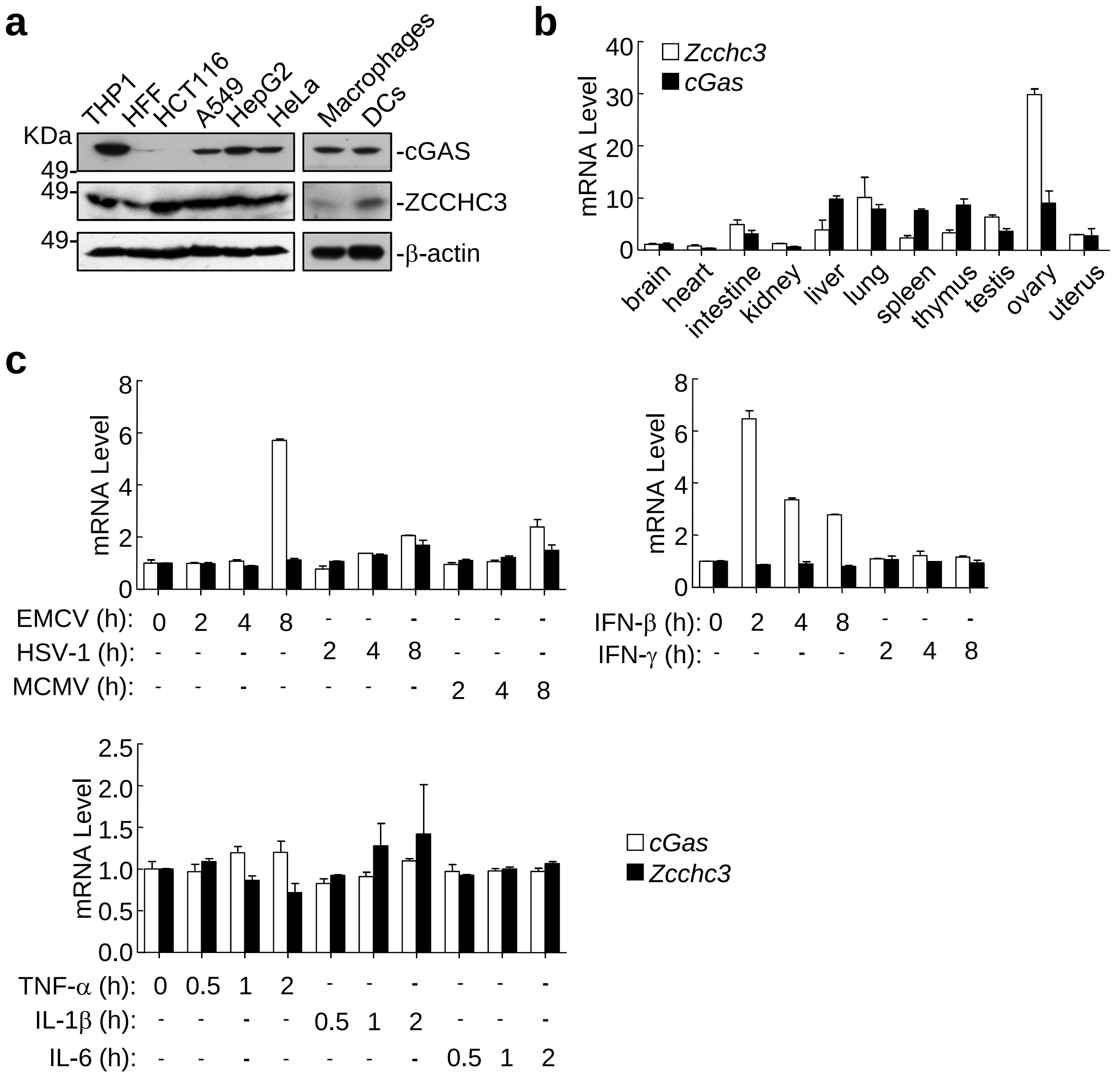

Supplementary Figure 4. Expression of ZCCHC3 and cGAS in different cells and tissues.
a Immunoblotting analysis of ZCCHC3 and cGAS levels in the indicated human cell lines. **b** qPCR analysis of mRNA levels of ZCCHC3 and cGAS in murine tissues. qPCR was performed with RNAs extracted from the indicated tissues of eight-week-old mice. **c** Effects of various stimuli on transcription of *ZCCHC3* and *cGAS*. MLFs were infected with the indicated viruses or treated with the indicated cytokines for the indicated times before qPCR analysis for ZCCHC3 and cGAS mRNA levels. Data are representative of two experiments with similar results.

Figure 1d

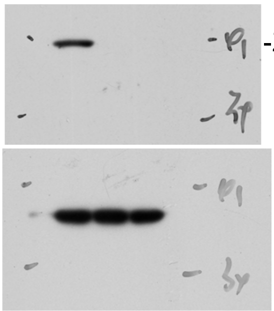

Figure 1f

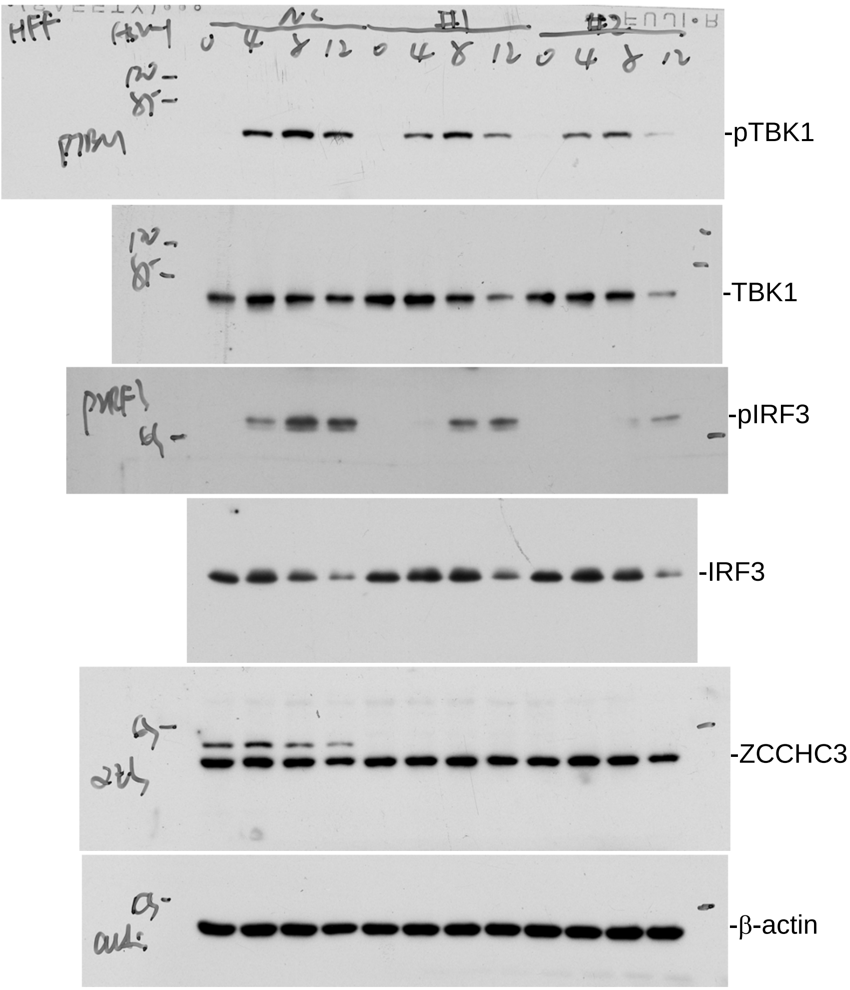

Figure 1h

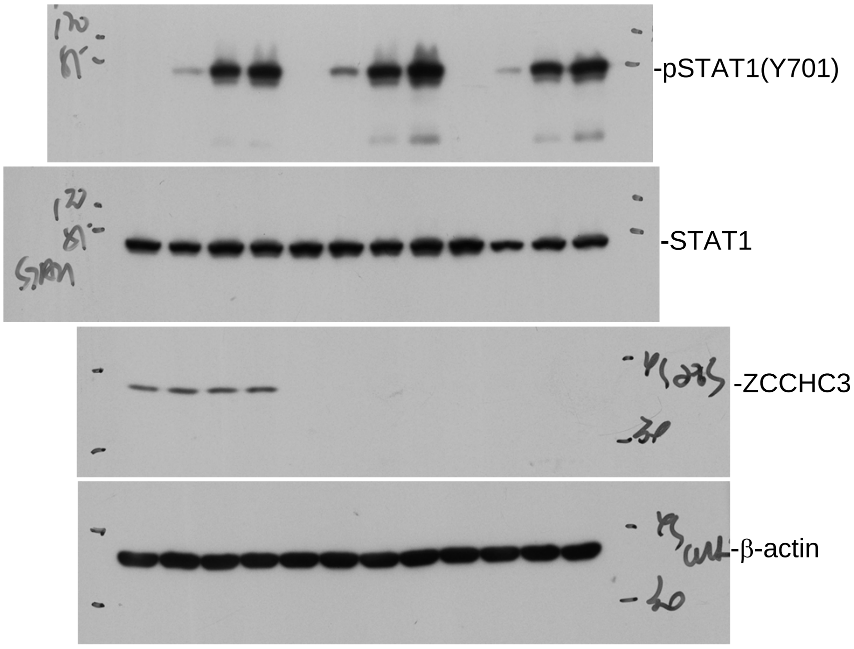

Supplementary Figure 5. Un-cropped immunoblots for Figure 1d, 1f and 1h.

Figure 2f

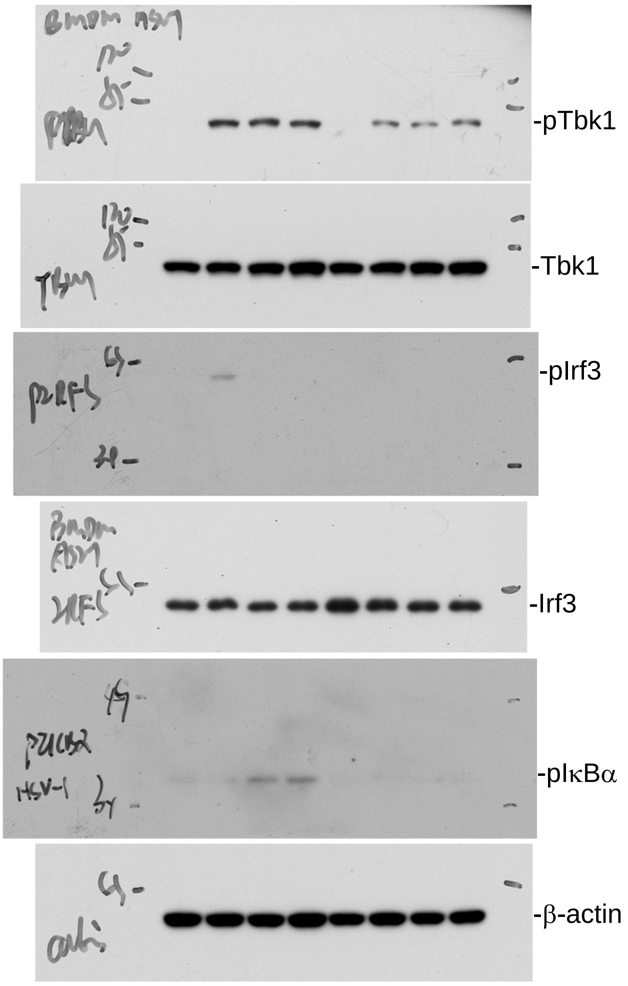

Supplementary Figure 6. Un-cropped immunoblots for Figure 2f.

Figure 4c

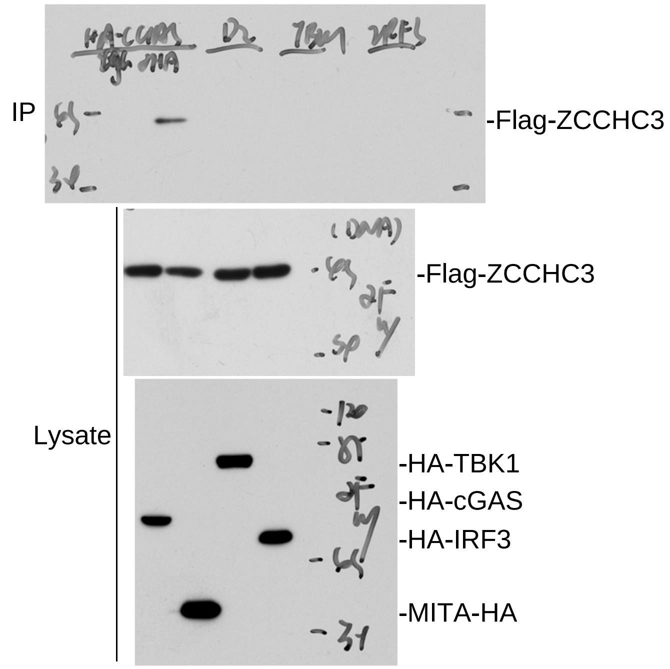

Figure 4f Left blots

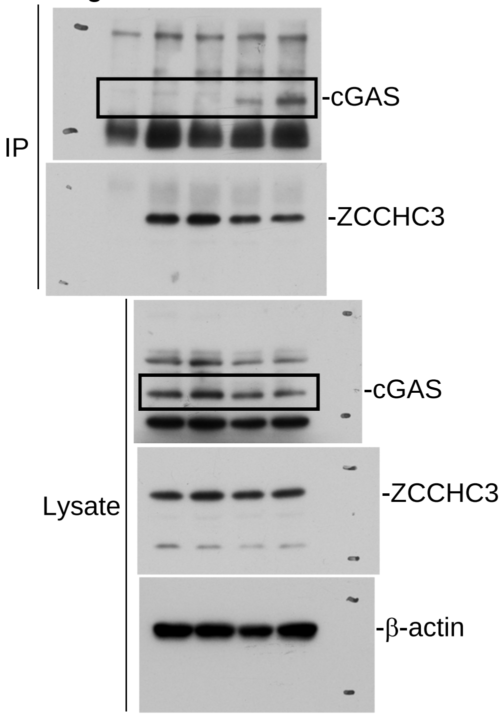

Figure 4f Right blots

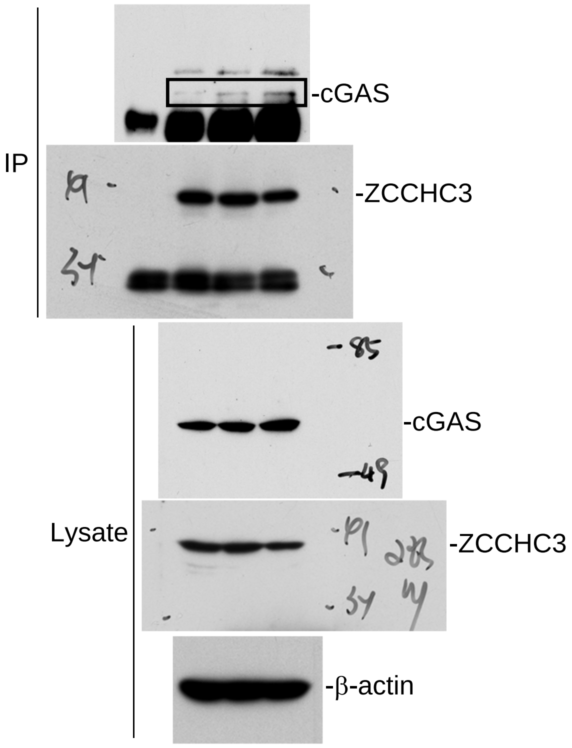

Supplementary Figure 7. Un-cropped immunoblots for Figure 4c and 4f.

Figure 6a

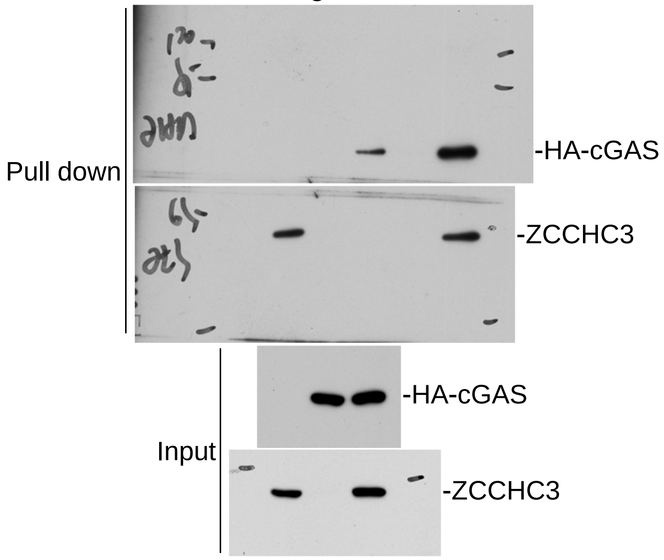

Figure 6b

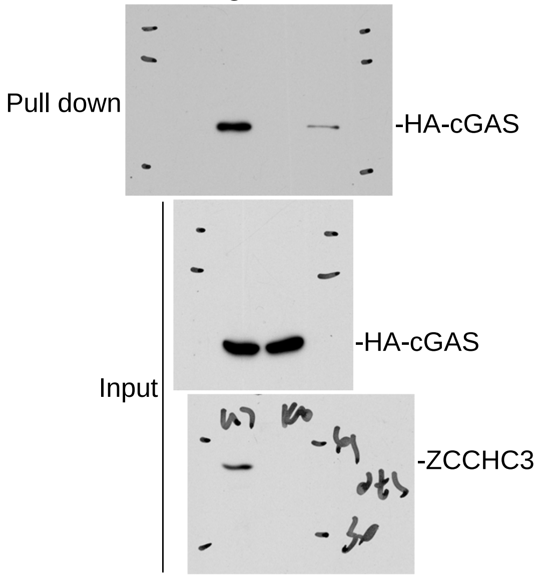

Figure 6c Left blots

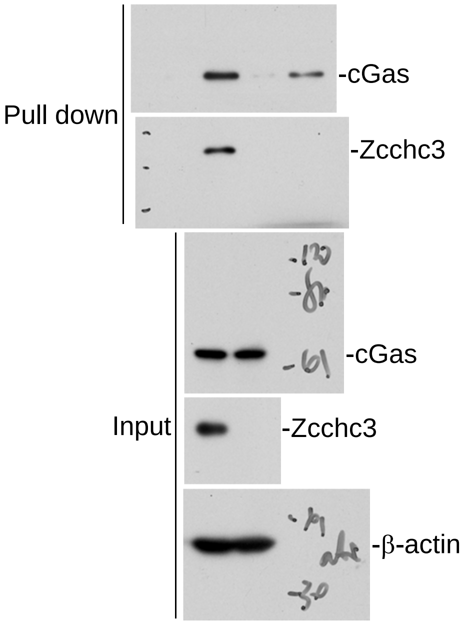

Figure 6c Right blots

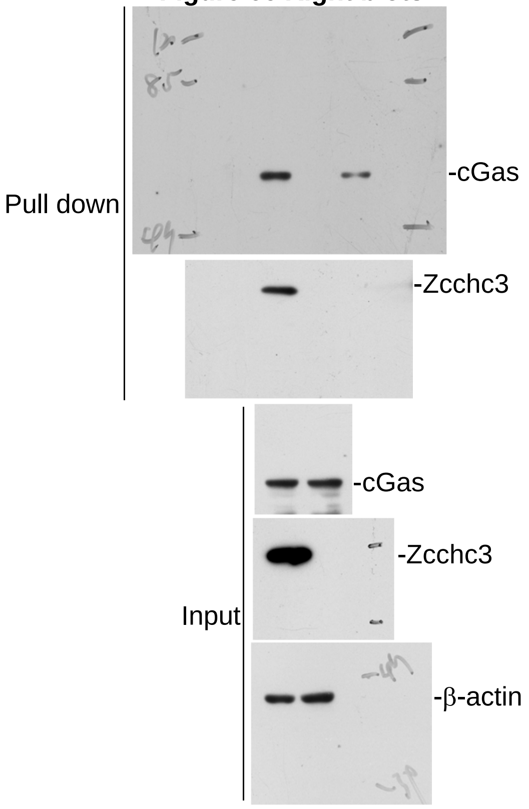

Supplementary Figure 8. Un-cropped immunoblots for Figure 6a, 6b and 6c

Figure 7a

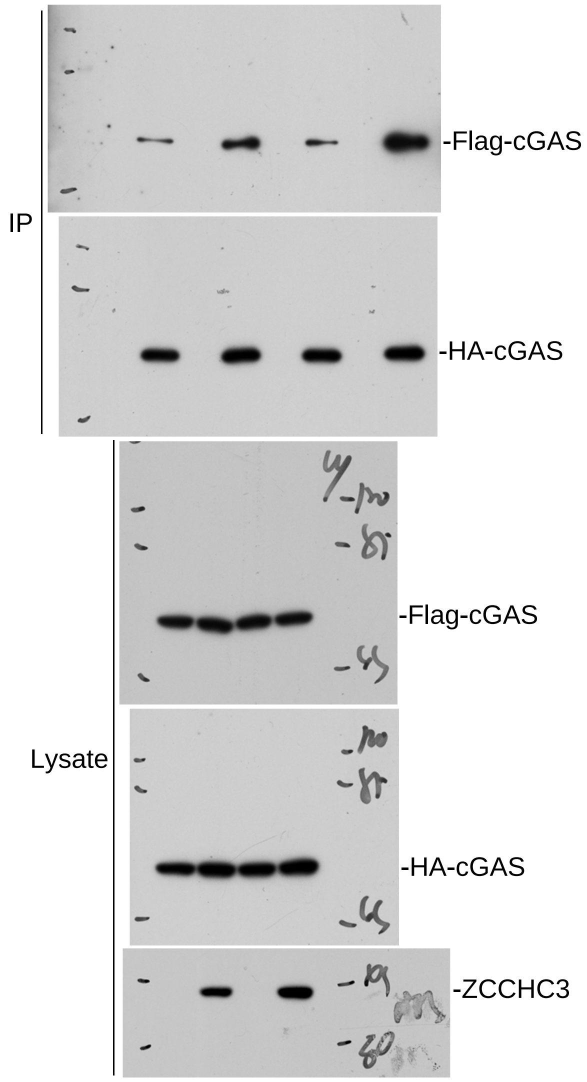

Figure 7b

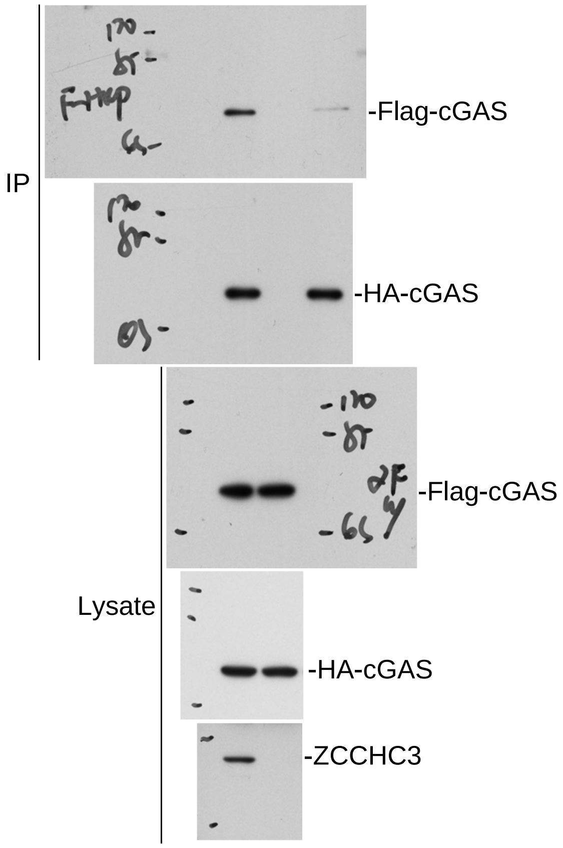

Figure 7c

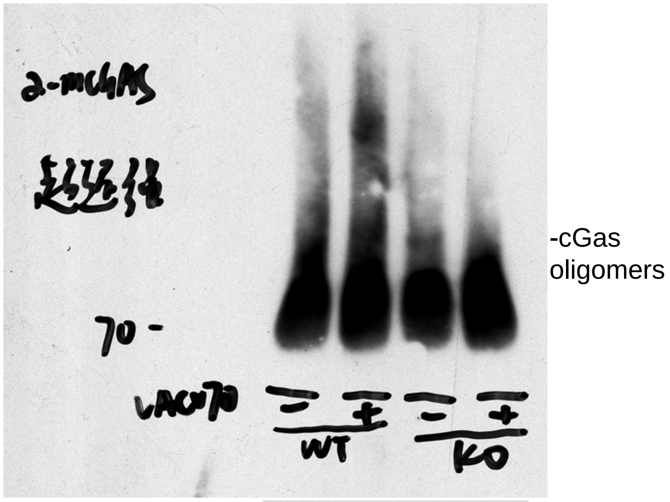

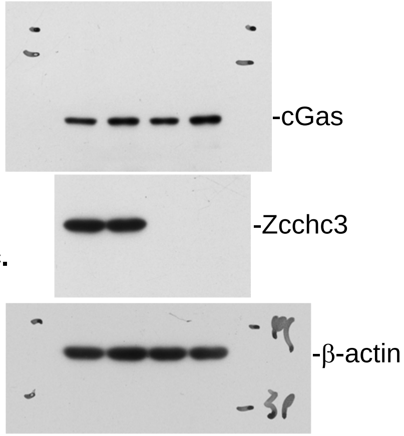

Supplementary Figure 9. Un-cropped immunoblots for Figure 7a, 7b and 7c.

Supplementary Figure 1a

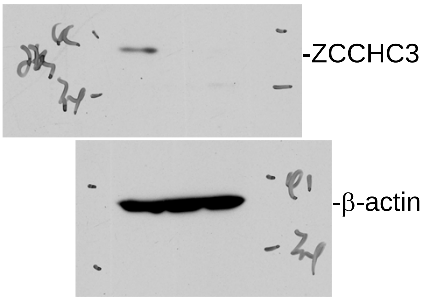

Supplementary Figure 1b

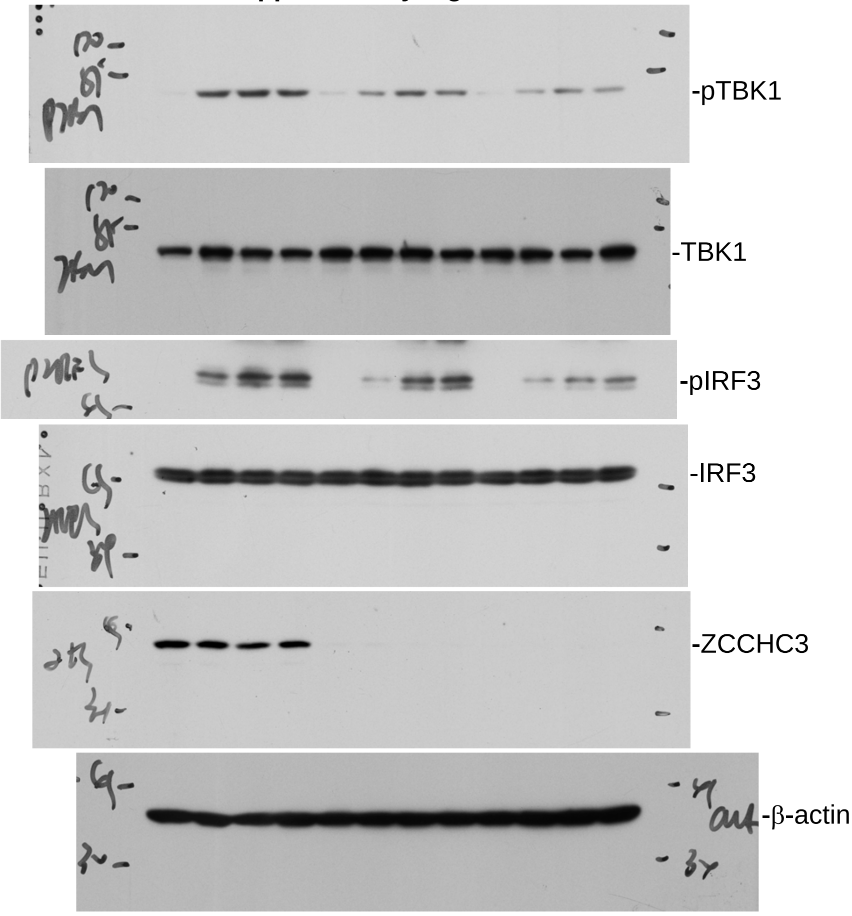

Supplementary Figure 10. Un-cropped immunoblots for Supplementary Figure 1a and 1b.

Supplementary Figure 2e

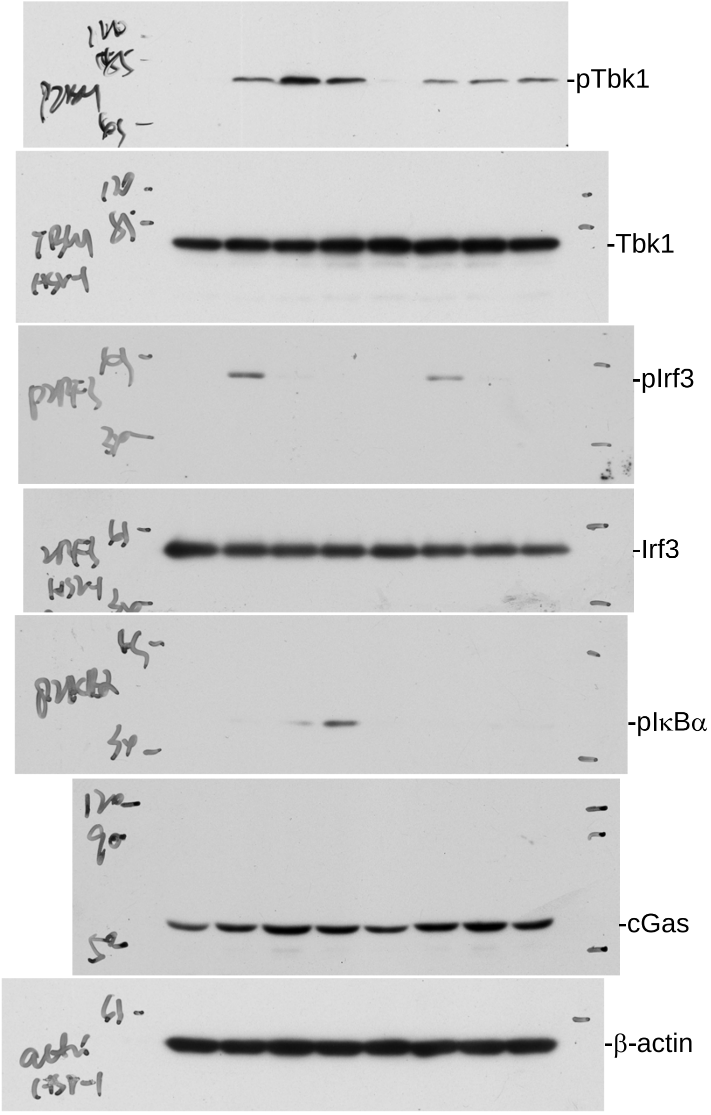

Supplementary Figure 11. Un-cropped immunoblots for Supplementary Figure 2e.

Supplementary Figure 3a

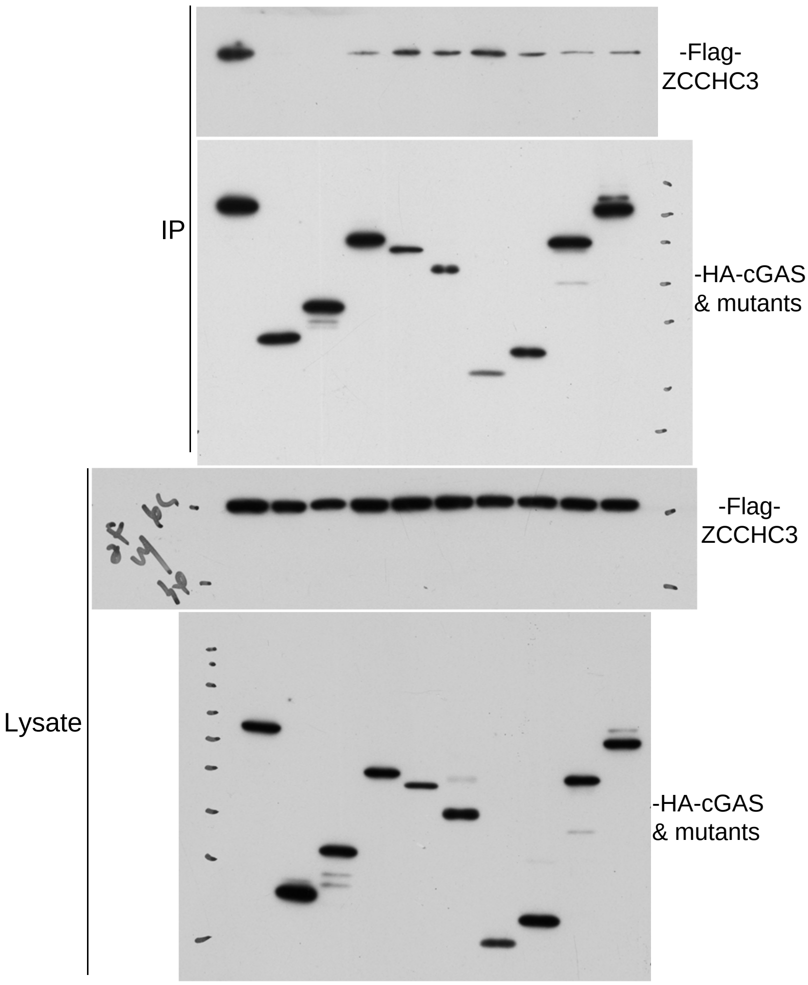

Supplementary Figure 3b

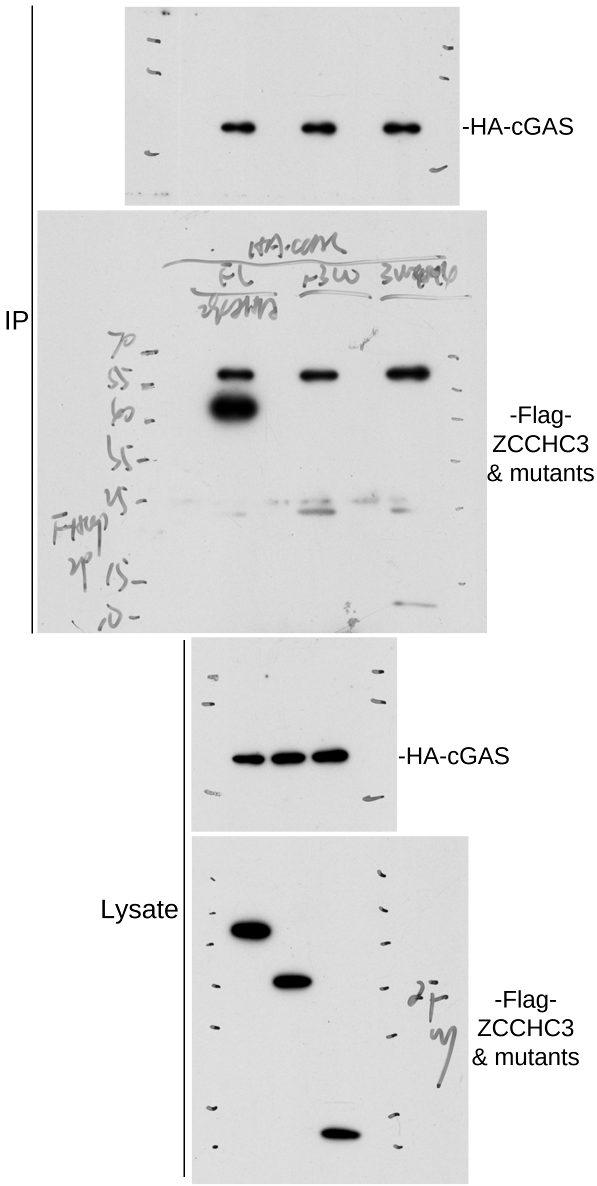

Supplementary Figure 4a

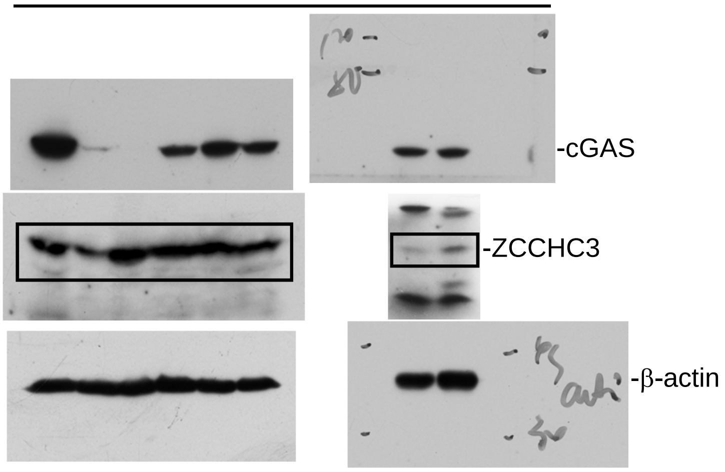

Supplementary Figure 12. Un-cropped immunoblots for Supplementary Figure 3a, 3b and Supplementary Figure 4a..

Table S1. Specific sequences of primers used in RT-PCR experiments

Gene	Forward	Reverse
GAPDH	GAGTCAACGGATTTGGTCGT	GACAAGCTTCCCGTTCTCAG
IFNB1	TTGTTGAGAACCTCCTGGCT	TGACTATGGTCCAGGCACAG
CXCL10	GGTGAGAAGAGATGTCTGAATCC	GTCCATCCTTGGAAGCACTGCA
ISG56	TCATCAGGTCAAGGATAGTC	CCACACTGTATTTGGTGTCTAGG
IL6	TTCTCCACAAGCGCCTTCGGTC	TCTGTGTGGGGCGGCTACATCT
RIG-I	ACGCAGCCTGCAAGCCTTCC	TGTGGCAGCCTCCATTGGGC
Gapdh	ACGGCCGCATCTTCTTGTGCA	ACGGCCAAATCCGTTCACACC
Ifnb1	TCCTGCTGTGCTTCTCCACCACA	AAGTCCGCCCTGTAGGTGAGGTT
Il6	TCTGCAAGAGACTTCCATCCAGTTGC	AGCCTCCGACTTGTGAAGTGGT
Cxcl10	ATCATCCCTGCGAGCCTATCCT	GACCTTTTTTGGCTAAACGCTTTC
Isg56	ACAGCAACCATGGGAGAGAATGCTG	ACGTAGGCCAGGAGGTTGTGCAT
Rig-I	AGCCAAGGATGTCTCCGAGGAA	ACACTGAGCACGCTTTGTGGAC
